# Making the Most of Its Short Reads: A Bioinformatics Workflow for Analysing the Short-Read-Only Data of *Leishmania orientalis* (Formerly Named *Leishmania siamensis*) Isolate PCM2 in Thailand

**DOI:** 10.3390/biology11091272

**Published:** 2022-08-26

**Authors:** Pornchai Anuntasomboon, Suradej Siripattanapipong, Sasimanas Unajak, Kiattawee Choowongkomon, Richard Burchmore, Saovanee Leelayoova, Mathirut Mungthin, Teerasak E-kobon

**Affiliations:** 1 Department of Genetics, Faculty of Science, Kasetsart University, Bangkok 10900, Thailand; 2 Omics Center for Agriculture, Bioresources, Food, and Health, Kasetsart University (OmiKU), Bangkok 10900, Thailand; 3 Department of Microbiology, Faculty of Science, Mahidol University, Bangkok 10400, Thailand; 4 Department of Biochemistry, Faculty of Science, Kasetsart University, Bangkok 10900, Thailand; 5 Glasgow Polyomics, College of Medical, Veterinary and Life Sciences, University of Glasgow, Glasgow G12 8QQ, UK; 6 Department of Parasitology, Phramongkutklao College of Medicine, Bangkok 10400, Thailand

**Keywords:** sequence read analysis, *Leishmania orientalis*, leishmaniasis, genomics, bioinformatics

## Abstract

**Simple Summary:**

Leishmaniasis is a parasitic disease caused by flagellated protozoa of the genus *Leishmania*. Multiple genome sequencing platforms have been employed to complete *Leishmania* genomes at the expense of high cost. This study proposes an integrative bioinformatic workflow for assembling only the short-read data of *Leishmania orientalis* isolate PCM2 from Thailand and produce an acceptable-quality genome for further genomic analysis. This workflow gives extensive information required for identifying strain-specific markers and virulence-associated genes useful for drug and vaccine development before a more exhaustive and expensive investigation.

**Abstract:**

Background: *Leishmania orientalis* (formerly named *Leishmania siamensis*) has been neglected for years in Thailand. The genomic study of *L. orientalis* has gained much attention recently after the release of the first high-quality reference genome of the isolate LSCM4. The integrative approach of multiple sequencing platforms for whole-genome sequencing has proven effective at the expense of considerably expensive costs. This study presents a preliminary bioinformatic workflow including the use of multi-step de novo assembly coupled with the reference-based assembly method to produce high-quality genomic drafts from the short-read Illumina sequence data of *L. orientalis* isolate PCM2. Results: The integrating multi-step de novo assembly by MEGAHIT and SPAdes with the reference-based method using the *L. enriettii* genome and salvaging the unmapped reads resulted in the 30.27 Mb genomic draft of *L. orientalis* isolate PCM2 with 3367 contigs and 8887 predicted genes. The results from the integrated approach showed the best integrity, coverage, and contig alignment when compared to the genome of *L. orientalis* isolate LSCM4 collected from the northern province of Thailand. Similar patterns of gene ratios and frequency were observed from the GO biological process annotation. Fifty GO terms were assigned to the assembled genomes, and 23 of these (accounting for 61.6% of the annotated genes) showed higher gene counts and ratios when results from our workflow were compared to those of the LSCM4 isolate. Conclusions: These results indicated that our proposed bioinformatic workflow produced an acceptable-quality genome of *L. orientalis* strain PCM2 for functional genomic analysis, maximising the usage of the short-read data. This workflow would give extensive information required for identifying strain-specific markers and virulence-associated genes useful for drug and vaccine development before a more exhaustive and expensive investigation.

## 1. Introduction

Leishmaniasis is a significant vector-borne zoonotic disease caused by flagellated protozoans of the order Trypanosomatidae and genus *Leishmania*. The disease occurs in over 98 countries, including countries in Asia, Africa, America, and Europe [1,2,3]. The number of new patients has increased annually to around 1.7 million people yearly [4]. Human leishmaniasis is classified into three forms: cutaneous leishmaniasis (CL), mucocutaneous leishmaniasis (MCL), and visceral leishmaniasis (VL). VL is the most severe form, affecting the liver, lymph node, and spleen. If the patient does not receive appropriate treatment, the fatality rate can be 100% within two years [5,6]. The CL, one of the most common forms of leishmaniasis, shows skin lesions and ulcerates at the site bitten by sandflies [7], while the MCL is a rare form associated with *Leishmania braziliensis* [8]. According to the Centers for Disease Control and Prevention (CDC), approximately 21 of the 30 *Leishmania* species cause human infection.

Leishmaniasis in Thailand was considered as an imported disease before 1999 [9,10]. However, after 1999, a new species of *Leishmania* was identified from a Thai patient with visceral leishmaniasis, named *Leishmania siamensis* [11] and later renamed *Leishmania orientalis* [12]. Several *L. orientalis* isolates were discovered in Thailand, including CU1, PCM1, PCM4, PCM5, and the Trang lineage isolate PCM2 [13]. *L. orientalis* infection was also found in other countries, such as the CL cases in horses in Europe [14,15] and Florida, USA [16]. Livestock, such as donkeys, cows, sheep, goats, and camels, can be a reservoir of leishmaniasis and could spread the infection more easily [17,18,19]. The sandfly is considered a significant vector of leishmaniasis in Thailand, predominantly *Sergentomyia gemmea* in the northern and southern areas of the country [20]. However, the situation of *Leishmaniasis* in Thailand is also challenging to estimate because the patients often show no symptoms. Monitoring the spread and mutation of *L. orientalis* will prepare for the future outbreak and emergence of new virulent strains. The previous study established the prevalence of coinfection with *Leishmania* among Thai HIV patients who attended the HIV clinic in Trang province. *Leishmania* infection was detected in nearly one-fourth of the 724 individuals using either direct agglutination test (DAT) or ITS1-PCR tests, and the dominant species identified in this investigation were *Leishmania martiniquensis* and *L. orientalis* [21]. The coinfection of *Leishmania* and HIV enhances the host immunological degradation, leading to treatment failure, a high incidence of recurrence, and a high fatality rate [22]. For leishmaniasis treatment, failure has been documented in patients treated with most anti-leishmaniasis drugs [23,24,25,26,27,28,29,30]. Amphotericin B (AmB) remains an effective drug with mitigated toxic effects after liposomal formulation [24].

The *Leishmania* genomes have shown several intriguing aspects, including (1) lack of large subtelomeric regions; (2) absence of C-5 DNA methylation but having a hypermodified nucleobase or base J which is unique to the kinetoplastids; and (3) unusual gene regulation, such as the absence of introns and trans-splicing of mRNAs from ~200 polycistronic transcription units (PTUs), compared with other trypanosomatids [31,32,33,34]. Chromosome numbers also vary among the *Leishmania* species: 36 chromosomes in *Leishmania infantum*, *Leishmania donovani*, and *Leishmania major* [35]; 35 chromosomes in *L. braziliensis* [33,36]; and 34 chromosomes in *Leishmania mexicana* [36]. Some cellular components of *Leishmania* are associated with virulence, i.e., glycoinositolphospholipids (GIPLs) [37], lipophosphoglycans (LPGs) [38], proteophosphoglycans (PPGs) [39], and the 11 kDa kinetoplastid membrane protein (KMP-11) [40]. These components contribute to the infection, invasion, and establishment of the mammalian host. Although the precise effect of these *Leishmania* components on clinical symptoms in mammalian hosts is unclear, there is evidence that these components affect *Leishmania*–host immune cell interactions [41].

Despite concerns about the genetic variation of *L. orientalis* and their public health impacts, genomic information of multiple *L. orientalis* isolates has gained attention. To date, three major genome sequencing platforms (Illumina, PacBio, and Oxford Nanopore) have been used to create the complete genomic data of several *Leishmania* species, including the genome of *L. major* strain Friedlin [31,42,43,44], *L. infantum* strain JPCM5 [33,42], *L. martiniquensis* strain LSCM1 [45,46,47], and a recent *L. orientalis* strain LSCM4 isolated from the northern province of Thailand [46,47,48], in exchange for considerable cost invested in the genome project. Questions have arisen on whether the new *Leishmania* species shall have their whole genomes decoded using all techniques at first glance. When there are several closely related genomes available, the assistance of bioinformatic analysis on the draft genome derived from only the short-read genome sequencing method would be enough to answer basic genomic questions on virulence, pathogenesis, and drug resistance. Therefore, this research aims to design a bioinformatics workflow for analysing the whole-genome short-read data of *L. orientalis* strain PCM2 isolated from the southern province of Thailand by optimising the assembly methods and maximising the data output. This workflow would give preliminary information required for further plans on the *Leishmania* genome sequencing with other methods.

## 2. Materials and Methods

### 2.1. Culture of Leishmania orientalis Isolate PCM2

*Leishmania orientalis* isolate PCM2 was maintained and provided by the Department of Parasitology, Phramongkutklao College of Medicine, Thailand. The promastigotes were grown at 26 °C in RPMI 1640-modified with 13.3 mM glutamine, 2.5 mM arginine, 0.3 mM cysteine, 1.7 mM glutamate, 62.1 mM proline, 0.6 mM ornithine, 3.8 mM glucose, 2.2 mM fructose, 5.1 mM malate, 2.8 mM α-ketoglutarate, 0.5 mM fumarate, 0.5 mM succinate, 25 mM HEPES, 50 µg/mL gentamicin, 2× MEM vitamins (Gibco, Grand Island, NY, USA), and 20% heat-inactivated fetal bovine serum (HIFBS, Gibco, Grand Island, NY, USA).

### 2.2. Genomic DNA Preparation

Genomic DNAs were prepared from a late logarithmic phase of promastigotes. The promastigote pellet was washed in ultrapure water and suspended in 1 mL of lysis buffer (10 mM Tris, 10 mM KCl, 10 mM MgCl_2_, 0.5 M NaCl, 2 mM EDTA, and 0.5% SDS) and 20 µL of Proteinase K solution (20 mg/mL). The samples were incubated at 56 °C for 30 min; then chloroform:isoamyl alcohol (24:1) was added one time to the sample volume, and the samples were gently shaken vigorously for 10 min. The samples were centrifuged at 10,000 rpm at room temperature for 10 min, and the upper aqueous phase was collected. RNAse (20 mg/mL) solution was added for 10 µL and incubated at room temperature for 3 min. After RNase treatment, one-time chloroform:isoamyl alcohol (24:1) was added, and the samples gently shaken vigorously before centrifugation at 10,000 rpm at room temperature for 10 min. The upper aqueous phase was collected, and DNAs in the upper aqueous phase were precipitated in 200 µL of 4 M ammonium acetate and 800 µL absolute ethanol at −70 °C overnight. The precipitated samples were centrifuged at 10,000 rpm at 4 °C for 10 min and washed with 70% ethanol twice. DNA samples were air-dried at room temperature for 30 min and suspended in TE buffer (10 mM Tris-HCl pH 8.0, 0.1 mM EDTA). The DNA quality and quantity were assayed by measuring absorbance at 260/280 nm using the Nanodrop (Thermo Fisher Scientific, Waltham, MA, USA) and at the absorbance of 260 nm. The genomic integrity was analysed by 1% agarose gel electrophoresis. The samples were kept at −70 °C before proceeding to the genome sequencing.

### 2.3. Quality Check and Processing of the Raw Sequence Reads

A paired-end read library (101 bp) was constructed for the whole-genome sequencing using the Illumina HiSeq2000 platform (Illumina, San Diego, CA, USA). The quality of the raw sequence reads was checked by FastQC Version 0.11.9 (http://www.bioinformatics.babraham.ac.uk/projects/fastqc/) (accessed on 7 June 2021) [49], and the raw sequence reads were processed through the filtering and trimming with the cut-off value of 20 using BBDuk in the BBTools pipeline (sourceforge.net/projects/bbmap/) (accessed on 7 June 2021).

### 2.4. Bioinformatic Workflow for Assembling the Genomic Reads of Leishmania orientalis Isolate PCM2 by Hybrid Methods and Salvaging the Unmapped Reads

**Figure 1:**
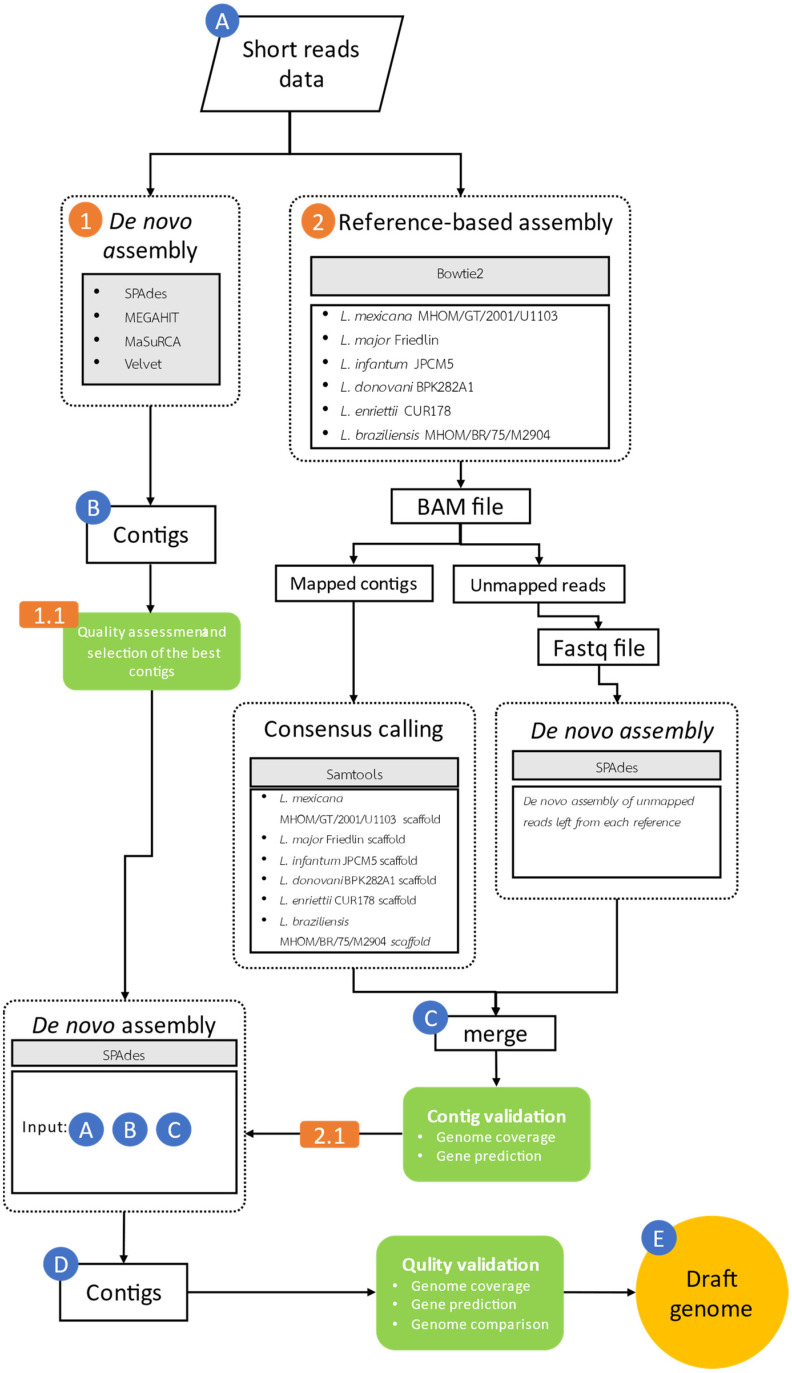
Diagram overview showing the Illumina read data management workflow, comparing the de novo method and the reference-based assembly.

This study designed a bioinformatics workflow for analysing the short-read genomic data of *Leishmania orientalis* isolate PCM2 as a preliminary overview which could be helpful to further decisions on the incorporation of additional sequencing platforms, as displayed in Figure 1. Initially, the filtered and trimmed reads (Data A in Figure 1) were processed separately through (1) de novo assembly and (2) referenced-based assembly and the de novo assembly of the remaining unmapped reads.

The first de novo assembly was conducted by using four assembly programs, namely SPAdes version 3.14 (Center for Algorithmic Biotechnology, St Petersburg, Russia) [50], MEGAHIT version 1.2.9 (HKU-BGI Bioinformatics Algorithms Research Laboratory & Department of Computer Science, L3 Bioinformatics Limited, Hong Kong, China) (National Institute of Informatics, Tokyo, Japan) [51], MaSuRCA version 4.0.5 (University of Maryland, College Park, MD, USA) [52], and Velvet version 1.2.10 (EMBL-European Bioinformatics Institute, Cambridge, UK) [53]. The assembled contigs (Data B in Figure 1) from each program were assessed by QUAST version 5.0.2 (Center for Algorithmic Biotechnology, St Petersburg, Russia) [54], and the best results were selected based on the contig length, the total number of contigs, and genome size (step 1.1). The Bowtie2 program version 2.4.2 (Johns Hopkins University, Baltimore, MD, USA) [55] performed the latter reference-based assembly using six complete genomes of *Leishmania* as references: *L. mexicana* MHOM/GT/2001/U1103 (GCA_000234665.4), *L. major* Friedlin (GCA_000002725.2), *L. infantum* JPCM5 (GCA_000002875.2), *L. donovani* BPK282A1 (GCA_000227135.2), *L. braziliensis* MHOM/BR/75/M2904 (GCA_000002845.2), and *Leishmania enriettii* CUR178 (GCA_017916305.1). A phylogenetic analysis of 443 partial sequences of heat shock protein 70-coding gene (*hsp*70) of *Leishmania* species and *Trypanosoma equiperdum* (an outgroup) downloaded from the NCBI nucleotide database was performed to select suitable reference genomes for this step. The sequences were trimmed to 1322 bp, multiply aligned by ClustalW version 2.1 (Conway Institute of Biomolecular and Biomedical Research, Belfield, Ireland) [56], and the phylogenetic relationship was reconstructed using neighbor-joining with the *p*-distance model and 10,000 bootstrap iterations using the MEGA X program (Research Center for Genomics and Bioinformatics, Tokyo, Japan) [57]. Although there was a chromosome-scale genome of another isolate LSCM4 of *L. orientalis* from the northern province of Thailand available, this genome was not incorporated into this process to resemble the situation in which no prior genomes of the same species were determined.

The quality of the mapped contigs from individual references was assessed by SAMtools version 1.14 (Wellcome Sanger Institute, Cambridge, UK) [58,59]. The unmapped reads from each reference-based assembly were de novo assembled by SPAdes version 3.14 (Center for Algorithmic Biotechnology, St Petersburg, Russia) [50]. These additional contigs were again combined with their corresponding mapped contigs, resulting in Data C in Figure 1. The obtained genomic contigs were subjected to gene prediction by AUGUSTUS Web Server [60,61,62]. The contigs from the two approaches (Data B and C) were integrated using the de novo assembler, SPAdes version 3.14 [50], using a parameter –trusted -contigs. Data A was then re-mapped to the integrated contigs using SPAdes, resulting in six genomic drafts (Data D).

### 2.5. Quality Examination of L. orientalis PCM2 Genomic Contigs by Genomic Comparison with the Reference Genomes

The contigs of *L. orientalis* isolate PCM2 (Data D in Figure 1) were mapped to seven associated reference genomes of *Leishmania* (*L. donovani* BPK282A1 (GCA_000227135.2), *L. braziliensis* MHOM/BR/75/M2904 (GCA_000002845.2), *L. infantum* JPCM5 (GCA_000002875.2), *L. major* Friedlin (GCA_000002725.2), *L. mexicana* MHOM/GT/2001/U1103 (GCA_000234665.4), *L. enriettii* CUR178 (GCA_017916305.1), and *L. orientalis* LSCM4 (GCA_017916335.1)) using Bowtie2 version 2.4.5 (Johns Hopkins University, Baltimore, MD, USA) [55] with their default parameters. Samtools version 1.14 (Wellcome Sanger Institute, Cambridge, UK) [58,59] was used to determine mapping statistics, including the total number of mapped, unmapped, and paired mapped reads, as well as further analysis of the alignment files. Samtools then converted the SAM file from Bowtie2 to the BAM format and sorted it [63]. Pairwise comparison of two *Leishmania* genomes was analysed using pairwise comparison methods using the Smith–Waterman algorithm [64], and the comparative dot plots were produced by the re-DOT-able tools (https://www.bioinformatics.babraham.ac.uk/projects/redotable/) (Babraham Institute, Cambridge, UK) (accessed on 7 June 2021). A coverage analysis on the *Leishmania* chromosomes was performed using Bowtie2. Calculation of the coverage analysis on each alignment was performed using SAMtools (SAMtools coverage). The visualization of our draft genome coverage against the seven *Leishmania* reference genomes was plotted and compared using the R package, karyoploteR library [65]. Genes were also predicted from Data D using the Augustus program [62,66] based on evidence of the protein homology to *L. major* Friedin (GCA_000002725.2). Gene ontology was assigned to the predicted protein-coding genes using Pannzer2 version 2 (University of Helsinki, Helsinki, Finland) [67,68,69,70,71,72]. The GO sets were simplified by grouping similar terms based on semantic similarity. The accuracy of the predicted GO class was estimated using positive predictive value (PPV). The relationship between PPV and the Argot score was calibrated using a training set of proteins with available correct annotation. The GO enrichment analysis was performed by using the enrichGO program from the enrichplot library for gene ontology over-representation test. Adjusted *p*-values for these multiple comparisons were obtained by using Benjamini and Hochberg methods by setting pvalueCutoff = 0.05 and qvalueCutoff = 0.10, and the enrichment results were plotted using the rrvgo library [73]. These parameters were used to select the best final draft genome (Data E in Figure 1).

## 3. Results

This study sequenced and reconstructed the draft genome of *L. orientalis* isolate PCM2, collected from the southern province in Thailand, exclusively based on the Illumina short-read data. A total of 16,980,871 sequence reads were generated and may frequently be considered insufficient for the current genomic research trend. The authors designed a bioinformatics workflow for use with these genomic reads as depicted in Figure 1 that (1) integrated de novo and reference-based assembly methods to handle novel reads and the reads that shared homology to the reference genomes; (2) de novo assembled by multiple assemblers; (3) recruited multiple reference genomes as choices; (4) employed multiple steps of the de novo assembly to assure that all reads would be involved in the draft genome; and (5) assessed the genome quality by judging the coverage, number of predicted genes, and the associated functional annotation. The first stage of de novo assembly compared the performance of four assemblers, namely SPAdes, MEGAHIT, MaSuRCA, and velvet (Table 1). The four assemblers gave contigs with similar GC contents (an average of 59.07%). MEGAHIT gave the genomic size of 29.94 Mb and contained the largest contigs of 85.32 Kb. Although the MEGAHIT-derived genomic size was slightly less than that of the SPAdes program, MEGAHIT achieved higher N50 and N70 values, representing 50% and 70% of the entire assembly larger than these values. Among these four assemblers, the performance of Velvet was the lowest, and that of MaSuRCA was second-lowest.

**Figure 2:**
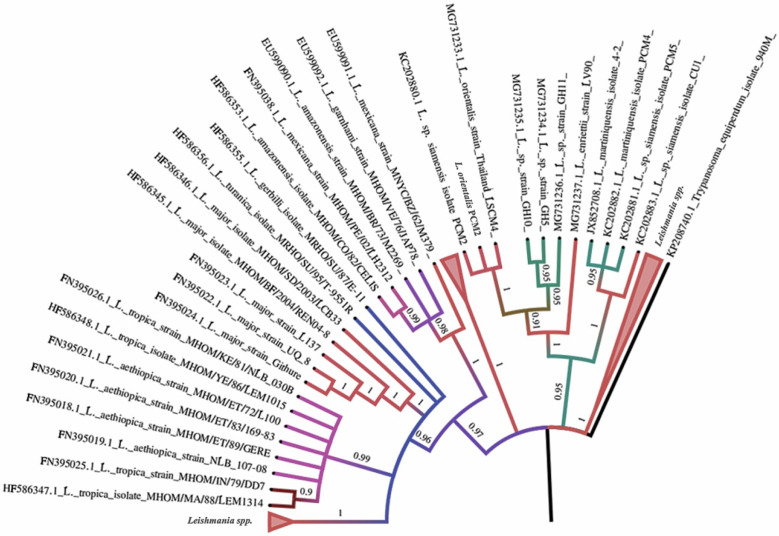
Phylogenetic relationship of *Leishmania* species reconstructed based on the heat shock protein 70 (hsp70) gene using the neighbour-joining method with the *p*-distance model and 10,000 bootstrap iterations. Line colours represent clusters of the samples. The branch number indicates the bootstrap value. The red triangles represent the distant clades which included sequences of *Leishmania infantum* (123 samples), *Leishmania donovani* (91 samples), *Leishmania braziliensis* (90 samples), *Leishmania tropica* (38 samples), *Leishmania major* (31 samples), *Leishmania turanica* (10 samples), *Leishmnia naiffi* (9 samples), *Leishmania chagasi* (6 samples), *Leishmnia aethiopica* (6 samples), *Leishmania* sp. (5 samples), and *Leishmania panamensis* (4 samples).

The hsp70 of *Leishmania orientalis* isolate PCM2 was retrieved from the MEGAHIT-derived contigs and was utilised to determine the evolutionary relationship among various *Leishmania* species. The phylogenetic tree clustered *L. orientalis* isolates PCM2 and LSCM4 (MG731233.1) and another PCM2 sample previously named *L. siamensis* (KC202880.1). The phylogenetic result suggested that *L. enriettii* was closely related to the *L. orientalis* PCM2 (Figure 2), consistent with prior analysis [74]. Therefore, the genome of *L. enriettii* was selected and used as the reference along with the other five complete *Leishmania* genomes. The reference-based assembly gave different genome sizes, numbers of contigs, and genes (Table 2). The longest length (33.32 Mb), highest N75 value (709,397 bases), and the highest number of predicted genes (8545 genes) were achieved by using *L. enriettii* CUR178 as the reference (marked as bold on the Scaffold (B) row in Table 2). This method yielded fewer contigs and larger contig sizes than the de novo assembly. The reference genomes of *L. donovani* BPK282A1 and *L. major* Friedlin in the assembly produced the lowest number of contigs (36 contigs), and the N50 value was the highest when using *L. major*. In comparison, the largest contig (3343,498 bases) was obtained when *L. mexicana* MHOM/GT/2001/U1103 was used as a reference. The de novo assembly of the remaining reads from the reference-guided mapping could provide additional contigs: the maximum of 12,929 contigs from the *L. donovani* dataset, the longest contig (33,294 bases) from the *L. infantum* and *L. major* datasets, the highest number of genes (3674 genes) from the *L. mexicana* dataset. To construct genomic scaffolds of *L. orientalis* isolate PCM2, the integrating multi-step de novo assembly with the reference-based method and salvage of the unmapped reads resulted in the lowest contig number (3367 contigs) and the highest number of predicted genes (8887 genes) for the *L. enriettii* dataset, yielding the largest scaffold of 30.27 Mb (marked as the bold red number in Table 2) at the expense of reduced length of the largest contig, N50, and N75 values. The results from the integrated approach were comparable to those of *L. orientalis* isolate LSCM4 (8158 genes) collected from the northern province of Thailand. Changes in the number of predicted genes were the main improvement of our proposed workflow compared to the use of the de novo assembly method or reference-based assembly alone which gave a broad range of the gene number, approximately 2265–7863 genes, with different contig numbers and lengths (Table 2).

**Figure 3:**
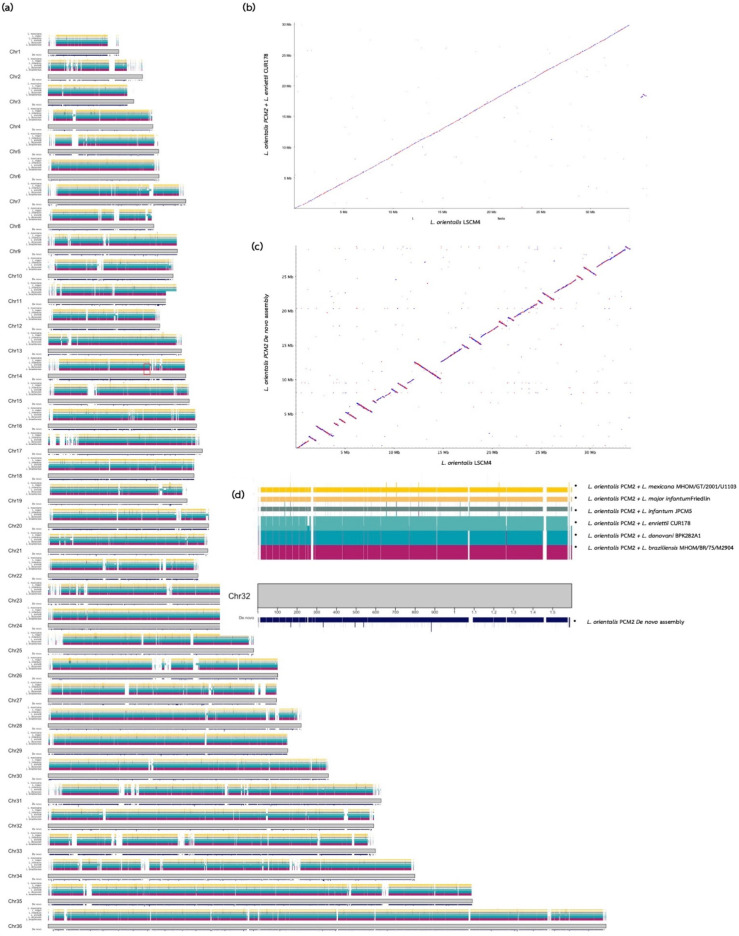
Comparative read mapping of the contigs of *L. orientalis* isolate PCM2 from different assembly methods: (1) a single de novo step; (2–7) six reference-based assemblies integrated with multi-step de novo assembly, and the salvage of the unmapped reads, with the chromosome-scale genome of *L. orientalis,* isolate LSCM4. The contigs assembled by six *Leishmania* reference genomes are shown in different colour strips. (**a**) Overview of genome alignments between these seven datasets with the LSCM4 chromosomes. Dot plots represent the high levels of synteny when comparing the *L. enriettii*-based contigs of the isolate PCM2 (**b**) and the single de novo assembled contigs of the PCM2 isolate (**c**) with the LSCM4 genome. (**d**) Example of regions on chromosome 32 with better genomic coverage of *L. enriettii*-based assembled contigs.

The contigs were mapped to the chromosomes of the closely related strain LSCM4 of *L. orientalis* for better visualisation of the assembled contigs (Figure 3a). The contigs from our integrating method with *L. enrietii* as a reference showed the best integrity and coverage of the assembly (Figure 3a and Figure S1) and better contig alignment to the genome of *L. orientalis* isolate LSCM4 (Figure 3b,c), indicating that the de novo assembly method alone may not be able to properly anticipate the contig direction. Our method also solved the problem of gap bridging that occurred during the de novo assembly process and can be seen as multiple gaps (Figure 3d) as examples in chromosomes 3, 4, 6–8, 11, and 13–36 in Figure S1. However, some large gaps remained in chromosomes 1–5, 7–12, 15, 17, 22–23, and 25–26 (in Figure S1), suggesting further low-depth long-read sequencing for closing these remaining gaps if researchers aim to complete these regions.

**Figure 4:**
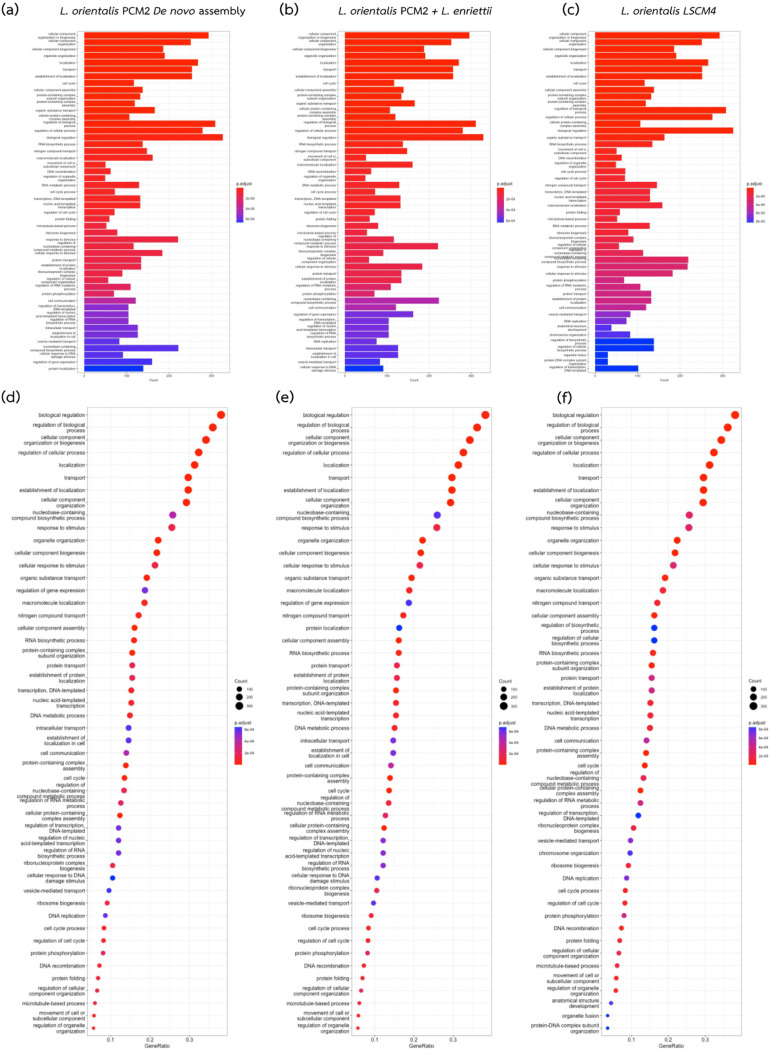
Distribution of the GO-annotated genes of the assembled genome of *L. orientalis* isolate PCM2 obtained from the single de novo assembly (**a**,**d**) and the integrated workflow (**b**,**e**) compared with the reference genome of *L. orientalis* isolate LSCM4 (**c**,**f**). (**a**–**c**) Plots between the GO term categories and gene count with their associated *p* values. (**d**–**f**) Plots between the GO terms and the gene ratios (proportion of all assigned GO functions in each genome) with their associated *p* values. The balloon sizes indicate the gene count.

Analysis of the annotated genes from *L. orientalis* strain PCM2 obtained by using our proposed method (Figure 4b,e) compared with the single de novo assembly (Figure 4a,d) and those of the reference *L. orientalis* strain LSCM4 (Figure 4c,f) showed similar patterns of gene ratios and frequency of each functional category. Fifty GO terms in the biological process category were assigned to the assembled genomes, and 23 of these (accounting for 61.6% of the annotated proteins) showed higher gene counts and ratios when the results from our workflow were compared to those of the LSCM4 isolate, i.e., biological regulation (GO:0065007), regulation of biological process (GO:0050789), cellular component organisation or biogenesis (GO:0071840), regulation of cellular process (GO:0050794), localisation (GO:0051179), transport (GO:0006810), establishment of localisation (GO:0051234), cellular component organisation (GO:0016043), nucleobase-containing compound biosynthetic process (GO:0034654), response to stimulus (GO:0050896), organelle organisation (GO:0006996), etc. Similarly, 21 GO terms from our workflow had greater gene counts and ratios compared to the single assembly method. Very few GO terms assigned to the results from the combined workflow showed a lower number of gene counts and ratios compared to the other two methods, including regulation of transcription (GO:0006355), nitrogen compound transport (GO:0071705), and transcription (GO:0006351). Moreover, the orthologous gene comparison between the PCM2 and LSCM4 strains revealed 7626 shared functional clusters, 29 clusters unique to the LSCM4, and 19 clusters unique to the PCM2 strain, confirming the difference between the two *L. orientalis* strains. These results indicated that our proposed bioinformatic workflow produced an acceptable-quality genome of *L. orientalis* strain PCM2 for functional genomic analysis.

## 4. Discussion

Several studies have shown that reconstructing a complete *Leishmania* genome requires multiple whole-genome sequencing methods, such as combining data from second- and third-generation sequencing technologies [75]. However, the expense of employing the third-generation sequencing platform might make it inaccessible to everyone. Dividing the genome sequencing project into phases would maximise data usage and be more cost-effective. The first phase could begin with either the short- or long-read sequencing method depending on relevant reference genomes and research objectives. This study started with the short-read sequencing method because of (1) the availability and quality of reference genomes from the related *Leishmania* species and (2) the focus on the gene function. In a similar genomic study on *Leishmania naiffi* and *Leishmania guyanensis*, the researchers reconstructed the draft genomes based on the short sequence reads and found additional genes compared to the previous related reference of *Leishmania braziliensis* strain M2904 [76]. Their short-read genomic draft allowed identification of novel genes, alteration of the TATE transposon, and the change in the gene copy number, consistent with different numbers of annotated genes in Figure 4 of the present study. If there were no appropriate references, multiple short-read sequencing with varying library sizes or the Oxford Nanopore long-read sequencing would be the choices for estimating the genomic landscape.

Analysis of the short-read outputs with the combination of reference-based and multi-step de novo assembly methods ensured that almost all reads would be incorporated into the draft genome of the isolate PCM2. Using multiple assemblers and reference genomes could maximise the chance of obtaining good genomic drafts using the best available assembling algorithms (MEGAHIT and SPAdes) and genomic guides using the genome of the closely related species *L. enriettii* in this study. This study also found that the de Bruijn graph-based MEGAHIT program was another good assembler for the *Leishmania* genome compared to SPAdes which was used several times in our workflow, consistent with the report that the MEGAHIT program could handle large and complex next-generation sequencing datasets [51]. In contrast, several recent chromosome-scale *Leishmania* genomes, including *L. orientalis* isolate LSCM4, were assembled using de novo assembly of the long-read MinION data as guidance for mapping the Illumina short reads [45]. In this study, our assembled genome of *L. orientalis* isolates PCM2 from the proposed analytic workflow on the short-read data shared highly similar genomic synteny with those of the LSCM4, as shown in Figure 3, implying the genome structural similarity between the selected reference, *L. enriettii*, and the LSCM4 genome. The critical success of this phase would also depend on the selection of the reference genome to obtain results comparable to the use of both long and short reads. The reference in this study also enhanced the investigation of genome structure compared between the PCM2 and LSCM4 isolates, as displayed in Figure 3b.

Although our workflow produced genomic drafts qualified for functional analysis, limitations were also addressed. Certain structures of the *Leishmania* genome could be complex to resolve by the short-read technology, such as duplicated genes and long tandem repeats, which may play a role in gene expression mechanisms [42,43]. Repetition and aneuploidy have challenged the assembly of *Leishmania* genomes [43,77,78,79,80]. These would be estimated by counting the read coverage as explained in [75], which estimated the aneuploidy of each chromosome (S) by 2 × di/dm from the short-read data, where di is the median depth of each chromosome and dm is the median depth of the whole genome. The gene copy number was calculated by dFG = S × dHG, where dFG is the full cell depth with somy effect and dHG was the average haploid depth per gene without the somy effect. To estimate the ploidy in this study, the long-read data would be essential for preparing the chromosomal outline onto which the short reads are mapped.

With the first phase results, researchers could manage the second phase of the genome project, whether accepting the genomic draft quality for the downstream analysis or applying additional sequencing platforms for the genome refinement and filling the genomic gaps. As shown in this study, the proposed workflow (Figure 1 and Figure S2) generated an acceptable genomic draft and a similar number of annotated genes compared to the reference genome of *L. orientalis* isolate LSCM4 (Figure 3 and Figure 4). These are useful for further functional comparative genomic analysis to identify core and accessory genes involved in virulence and pathogenesis, such as genes involved in establishing localisation. Our workflow could also be extended to explore more genomes of related *Leishmania* variants and species with cost-effective expenditure, particularly in low-income and developing countries.

## 5. Conclusions

The proposed bioinformatics workflow, which employs the usage of multiple assemblers and genomic references and combines multi-step de novo assembly with the reference-based method and the salvage of the unmapped reads, could maximise the use of the short-read data of *L. orientalis* strain PCM2 with the genome structure and number of annotated genes comparable to those of the strain LSCM4. This workflow would give preliminary information required for further plans to expand the *Leishmania* genome sequencing project to cover more isolates and incorporate other sequencing methods.

## Figures and Tables

**Table 1:** Summary of the de novo assembly parameters after assembling sequence reads of *L. orientalis* isolate PCM2 using four assemblers.

Features	Sequence Assemblers			
	SPAdes	MEGAHIT	MaSuRCA	Velvet
Total base in the assembly (Mb)	30.15	29.94	28.50	29.05
No. of contigs	5565	6470	11,241	18,409
Largest contigs (kb)	60.19	85.32	33.26	27.81
N50	12,259	13,737	4883	2919
N75	6229	6714	2547	1450
%GC content	59.02	59.07	59.15	59.03

**Table 2:** Comparative assembly parameters of *L. orientalis* isolate PCM2 obtained from different assembling strategies: a single de novo step (A), the reference-based assembly alone with six reference genomes of *Leishmania* (B), the de novo assembly of the unmapped reads (C), and the multi-step de novo assembly and referenced-based method (D), compared with the reference genome of another *L. orientalis* isolate LSCM4. Bold numbers represent the maximum numbers for the (B) method. Underlined numbers represent the maximum numbers for the (C) method. Red numbers represent the maximum numbers for the (D) method.

Assembly Methods		Genes	Contigs	Largest Contigs	Total Length (Mb)	N50	N75	L50	L75	GC (%)
MEGAHIT (De novo assembly)	Scaffold (A)	7653	4989	90,086	29.94	11,804	5994	767	1663	59.03
*L. braziliensis* MHOM/BR/75/M2904	Scaffold (B)	2265	138	2,686,643	32.07	992,961	641,930	11	22	58.68
	Unmapped (C)	3670	12,897	30,803	13.17	967	683	3112	7219	60.02
	Merged (D)	8187	5001	99,540	30.09	17,861	8663	**500**	**1100**	59
*L. donovani* BPK282A1	Scaffold (B)	2977	**36**	2,713,248	32.44	1,024,085	671,483	11	21	59.22
	Unmapped (C)	3672	12,929	30,803	13.17	968	683	3144	7250	60.02
	Merged (D)	8174	4973	99,540	30.08	18,120	8651	492	1087	59
*L. enriettii* CUR178	Scaffold (B)	**7863**	54	2,730,217	**33.32**	1,075,649	**709,397**	11	21	59.77
	Unmapped (C)	944	12,331	10,814	9.84	798	627	4427	7922	58.51
	Merged (D)	**8887**	**3367**	**206,463**	**30.27**	**24,513**	**13,215**	359	779	59.05
*L. infantum* JPCM5	Scaffold (B)	3101	76	2,673,956	32.12	1,043,848	659,512	11	21	59.29
	Unmapped (C)	3668	12,910	33,294	13.17	968	683	3133	7235	60.02
	Merged (D)	8181	4982	99,540	30.08	18,053	8696	498	1095	59
*L. major* Friedlin	Scaffold (B)	3030	**36**	2,682,151	32.86	**1,091,540**	684,829	11	21	59.31
	Unmapped (C)	3670	12,928	33,294	13.17	967	682	3135	7247	60.02
	Merged (D)	8178	4995	99,540	30.09	17,924	8632	495	1095	59
*L. mexicana* MHOM/GT/2001/U1103	Scaffold (B)	2930	575	**3,343,498**	32.10	1,044,075	655,046	10	20	59.41
	Unmapped (C)	3674	12,924	22,679	13.17	968	683	3140	7246	60
	Merged (D)	8184	4984	99,540	30.09	18,053	8615	496	1093	59
*L. orientalis* LSCM4	Scaffold (E)	8158	98	2,735,713	34.19	1,120,138	682,718	11	22	59.72

## Data Availability

None.

## Supplementary Materials

The following supporting information can be downloaded at: https://www.mdpi.com/article/10.3390/biology11091272/s1, Figure S1: Mapping the contigs of *Leishmania orientalis* strain PCM2 from the integrating assembly method based on six reference genomes of *Leishmania* and the single de novo method to the individual chromosomal sequences of *L. orientalis* isolate LSCM4. Figure S2: Graphical summary of the proposed analytic workflow based on the short-read data. The reads were assembled by the integration reference-based and multi-step de novo assembly.
