# Supplementary material for: Making the Most of Its Short Reads: A Bioinformatics Workflow for Analysing the Short-Read-Only Data of Leishmania orientalis (Formerly Named Leishmania siamensis) Isolate PCM2 in Thailand
Source: Biology (Basel). 2022 Aug 26;11(9):1272. doi: 10.3390/biology11091272 (PMC9495971; doi:10.3390/biology11091272)
Supplement: Supplementary file 1 [file biology-11-01272-s001.zip › Figure S1.pdf]

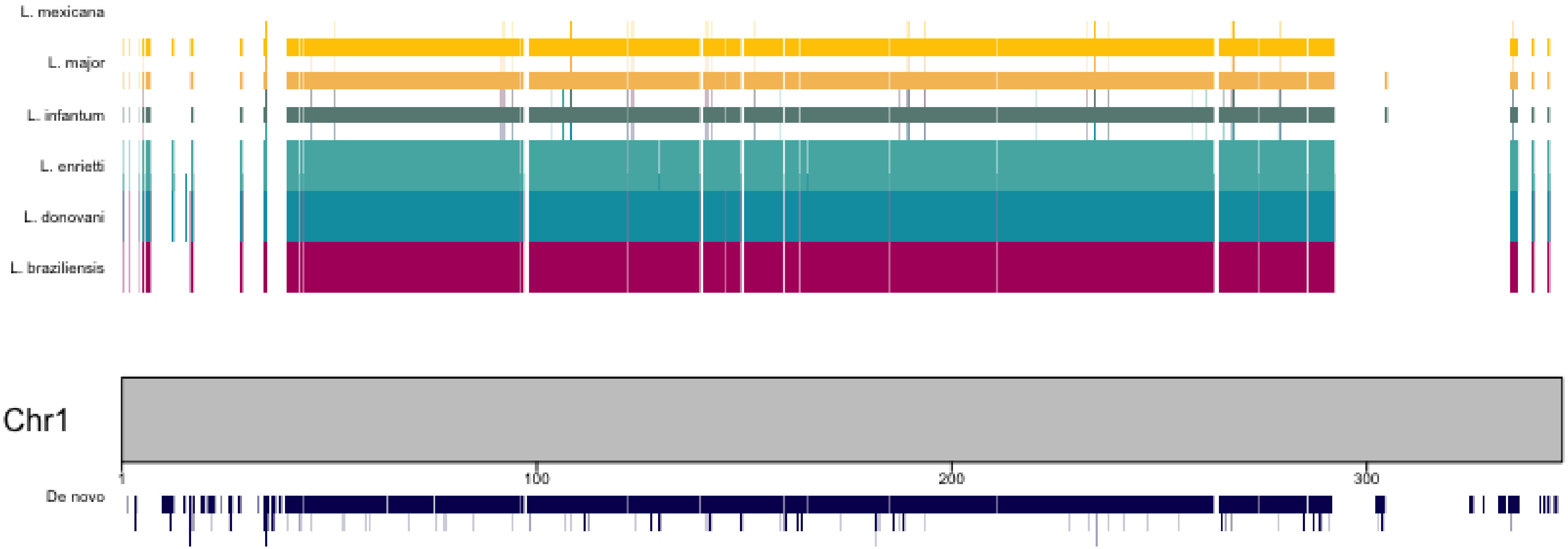

L. mexicana
L. major
L. infantum
L. enrietti
L. donovani
L. braziliensis
Chr1
1
100
200
300
De novo

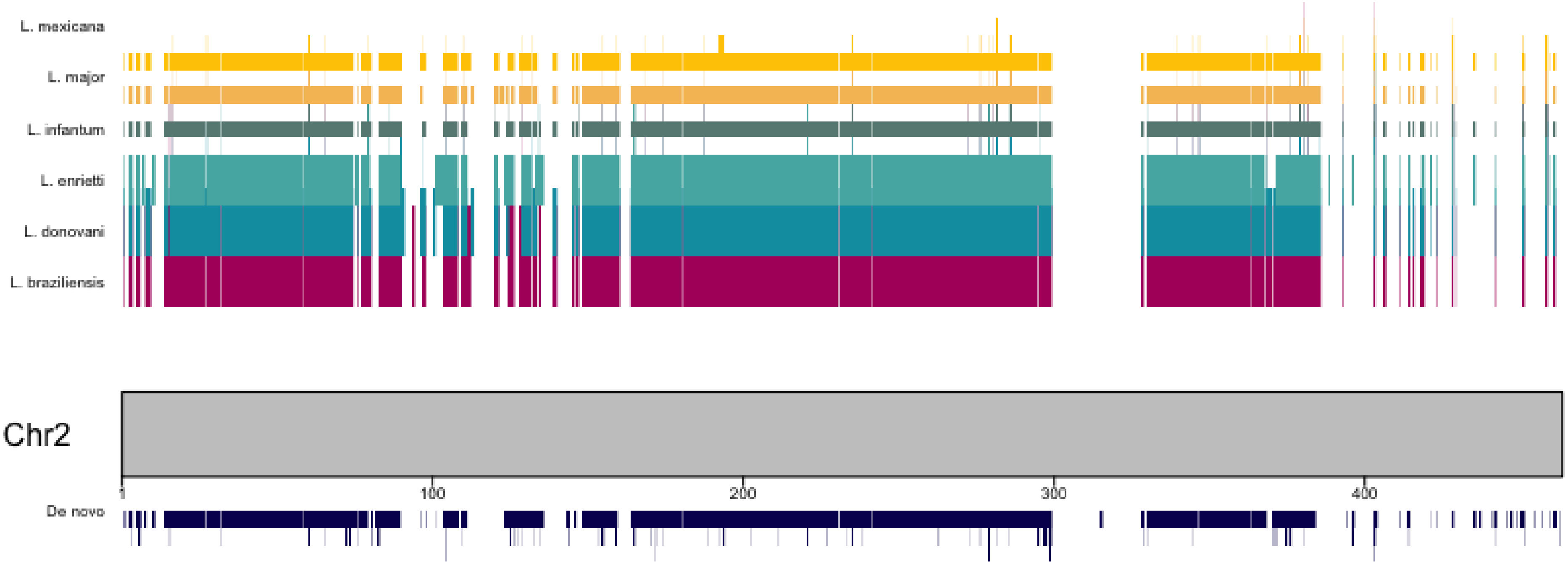

L. mexicana
L. major
L. infantum
L. enrietti
L. donovani
L. braziliensis
Chr2
1
100
200
300
400
De novo

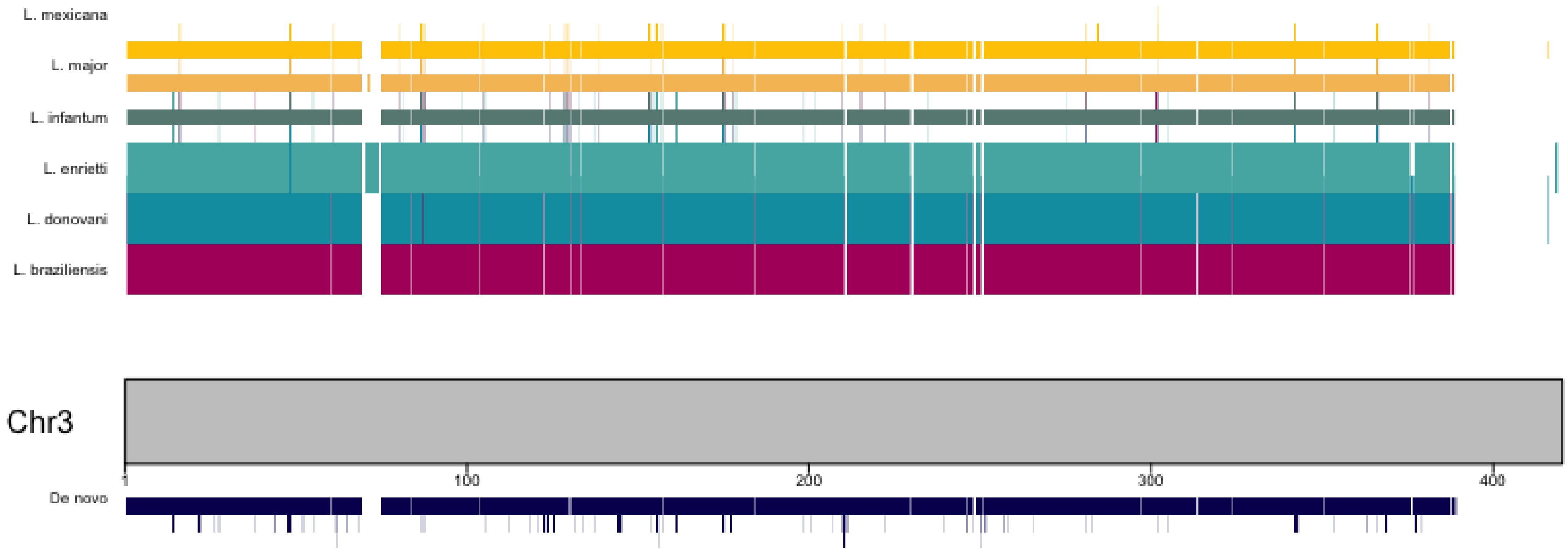
L. mexicana
L. major
L. infantum
L. enrietti
L. donovani
L. braziliensis
Chr3
1
100
200
300
400
De novo

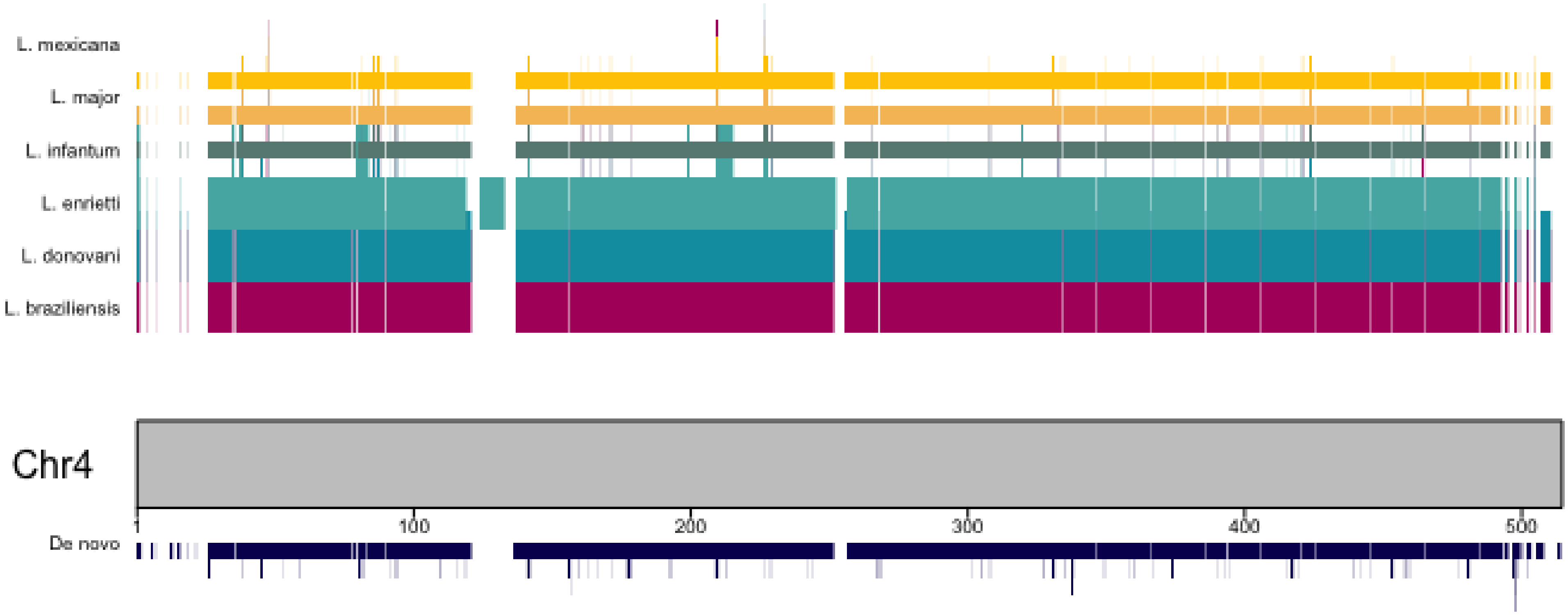

L. mexicana
L. major
L. infantum
L. enriettii
L. donovani
L. braziliensis
Chr4
1
100
200
300
400
500
De novo

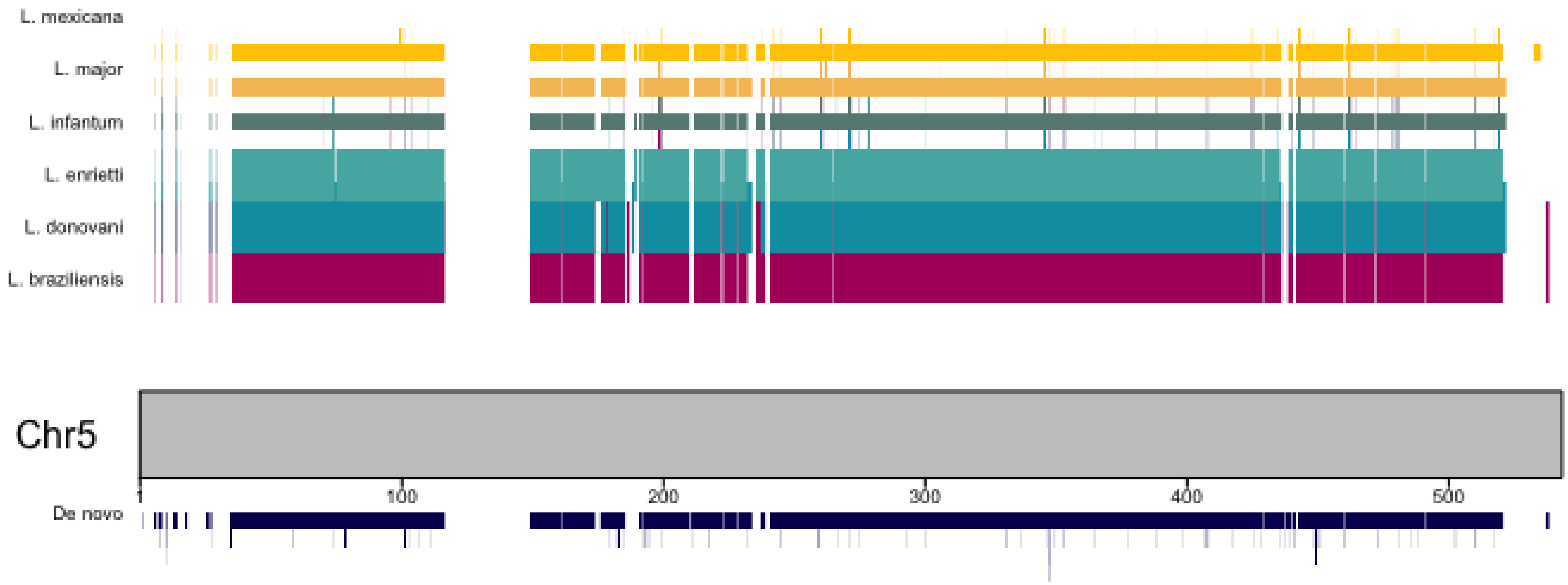
L. mexicana
L. major
L. infantum
L. enriettii
L. donovani
L. braziliensis
Chr5
1
100
200
300
400
500
De novo

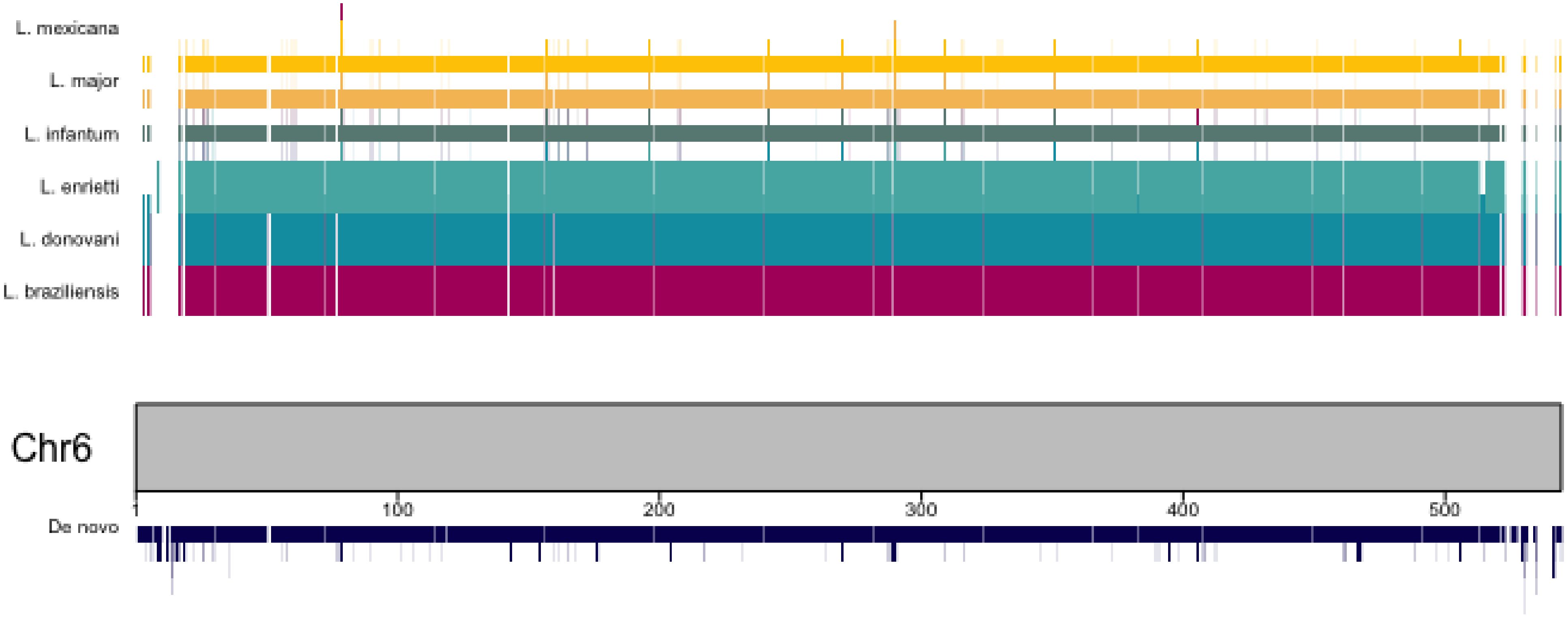

L. mexicana
L. major
L. infantum
L. enrietti
L. donovani
L. braziliensis
Chr6
1
100
200
300
400
500
De novo

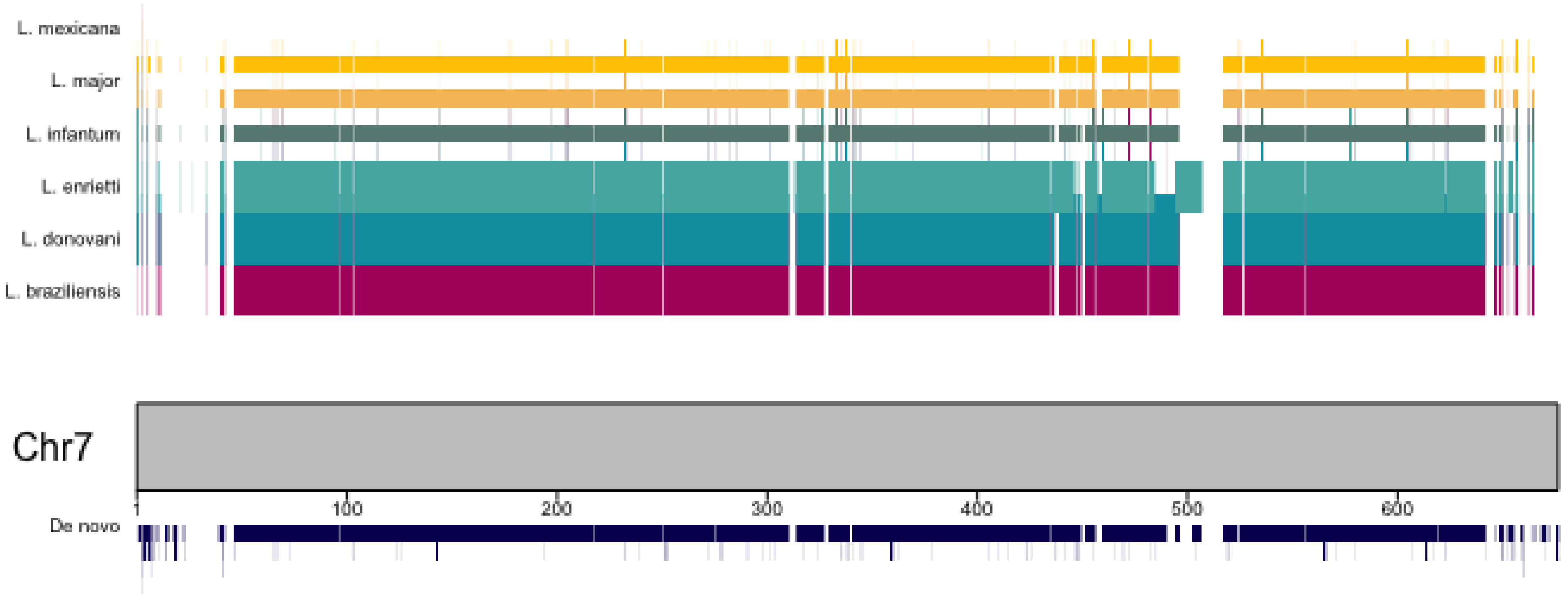

L. mexicana
L. major
L. infantum
L. enrietti
L. donovani
L. braziliensis
Chr7
1
100
200
300
400
500
600
De novo

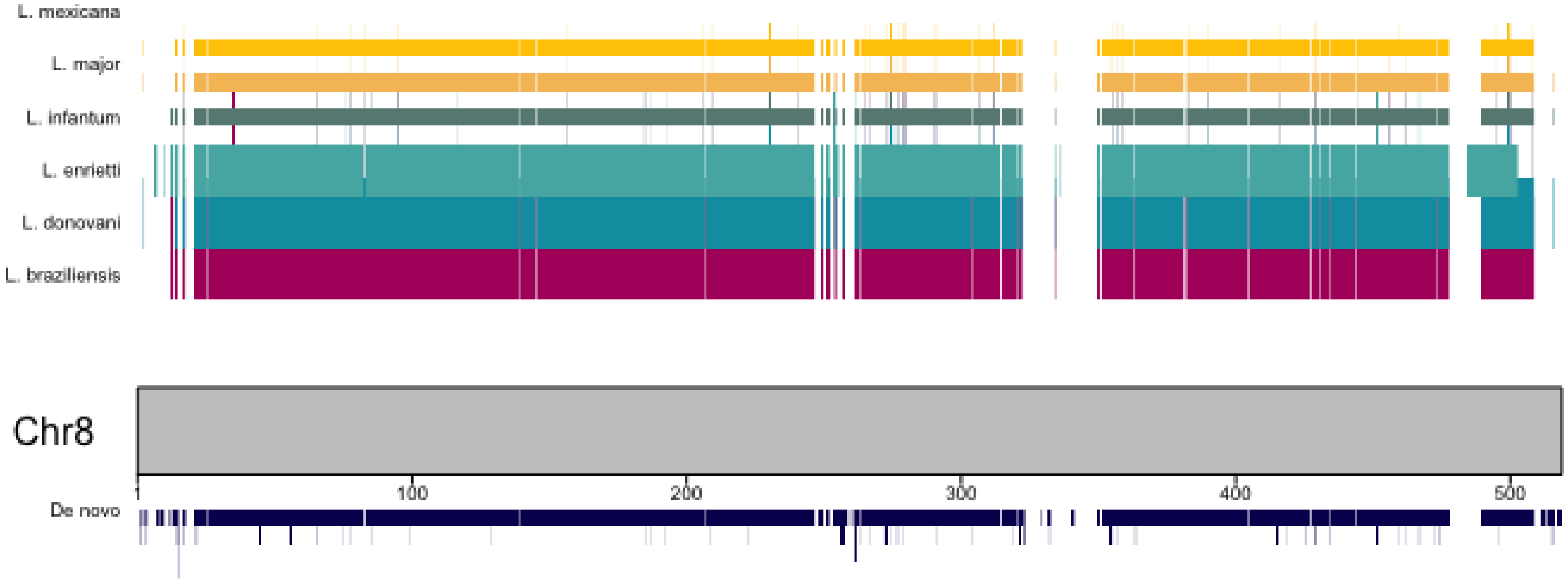
L. mexicana
L. major
L. infantum
L. enrietti
L. donovani
L. braziliensis
Chr8
1
100
200
300
400
500
De novo

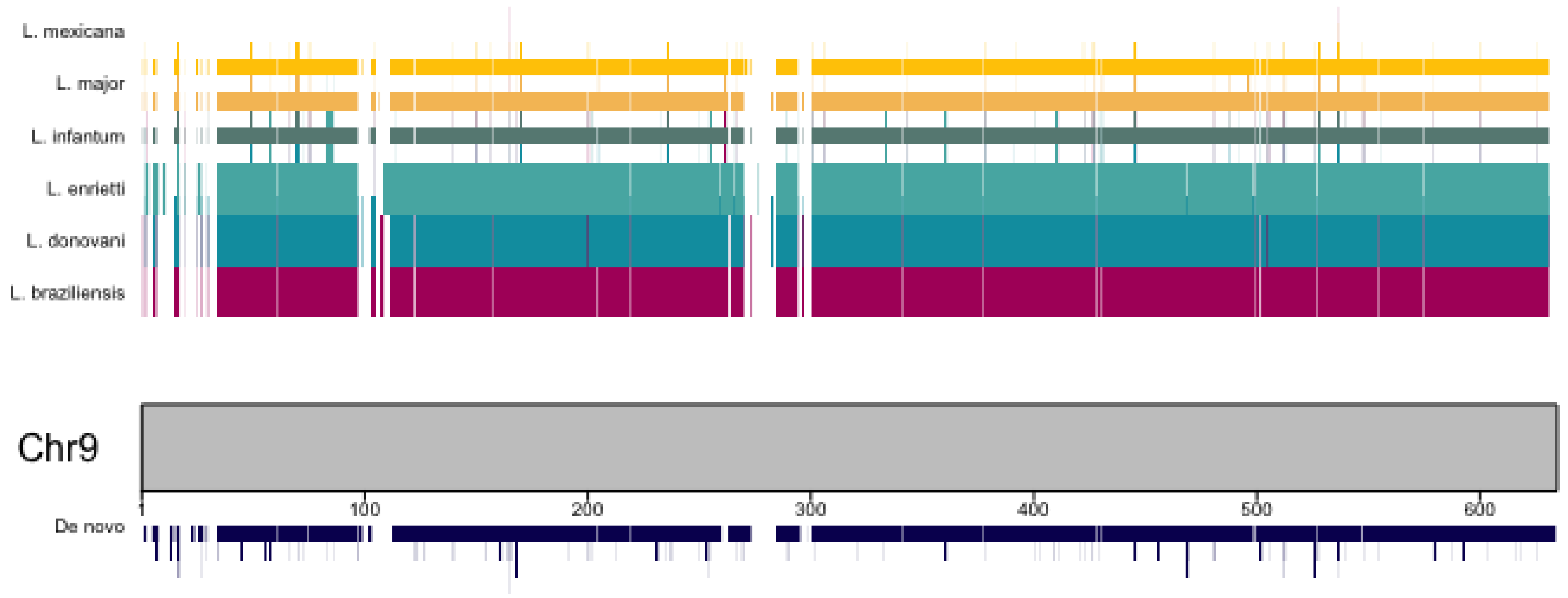
L. mexicana
L. major
L. infantum
L. enriettii
L. donovani
L. braziliensis
Chr9
1
100
200
300
400
500
600
De novo

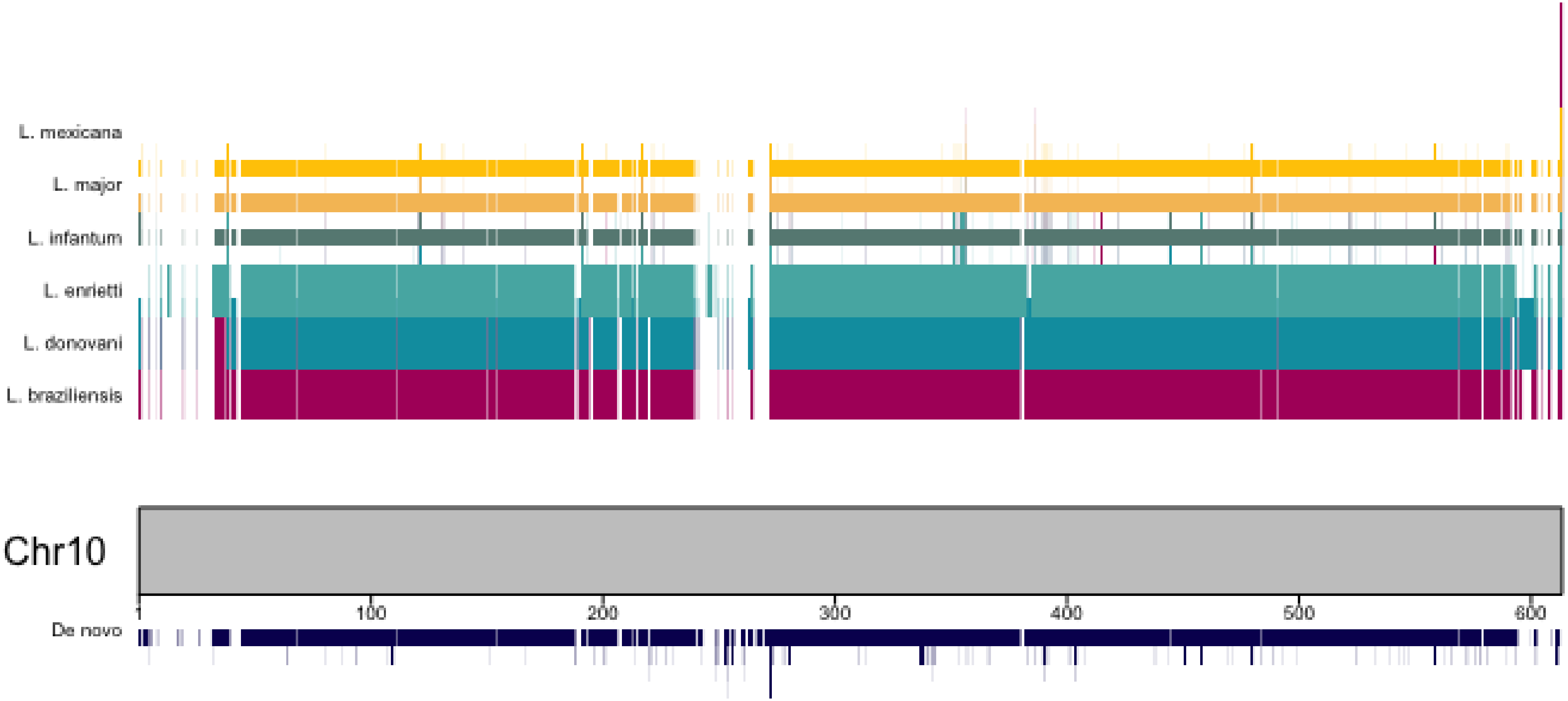
L. mexicana
L. major
L. infantum
L. enrietti
L. donovani
L. braziliensis
Chr10
1
100
200
300
400
500
600
De novo

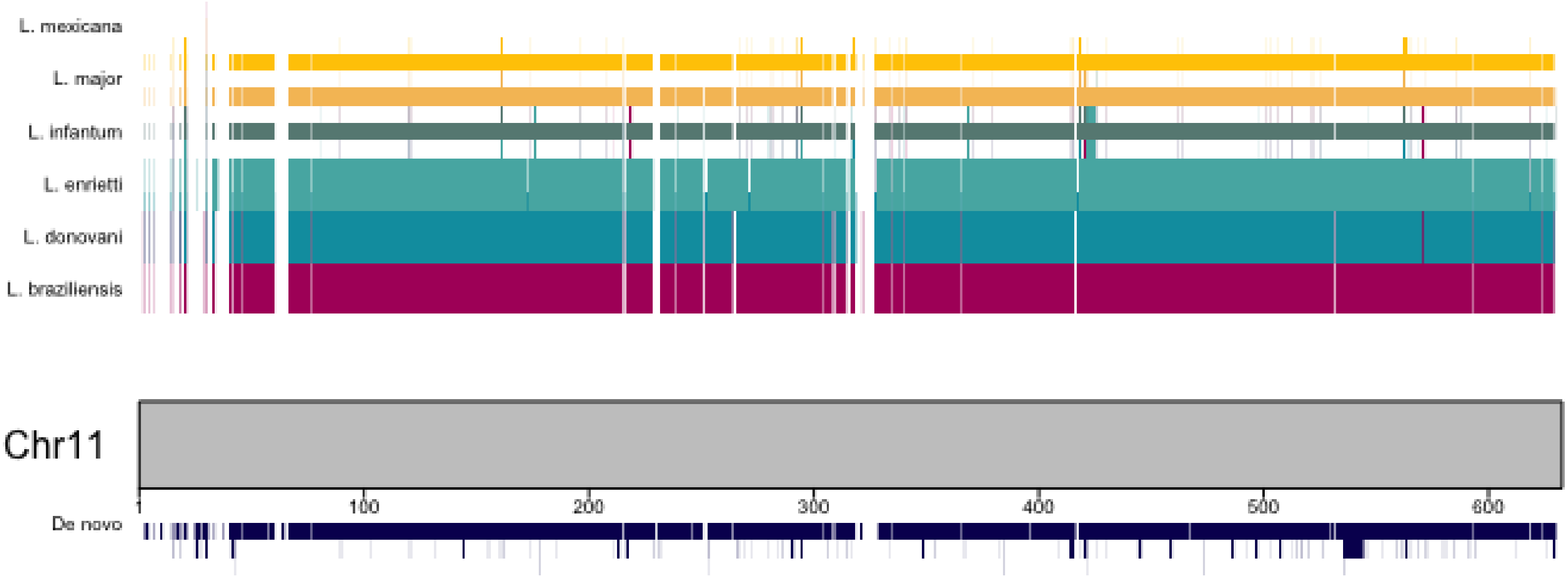

L. mexicana
L. major
L. infantum
L. enrietti
L. donovani
L. braziliensis
Chr11
1
100
200
300
400
500
600
De novo

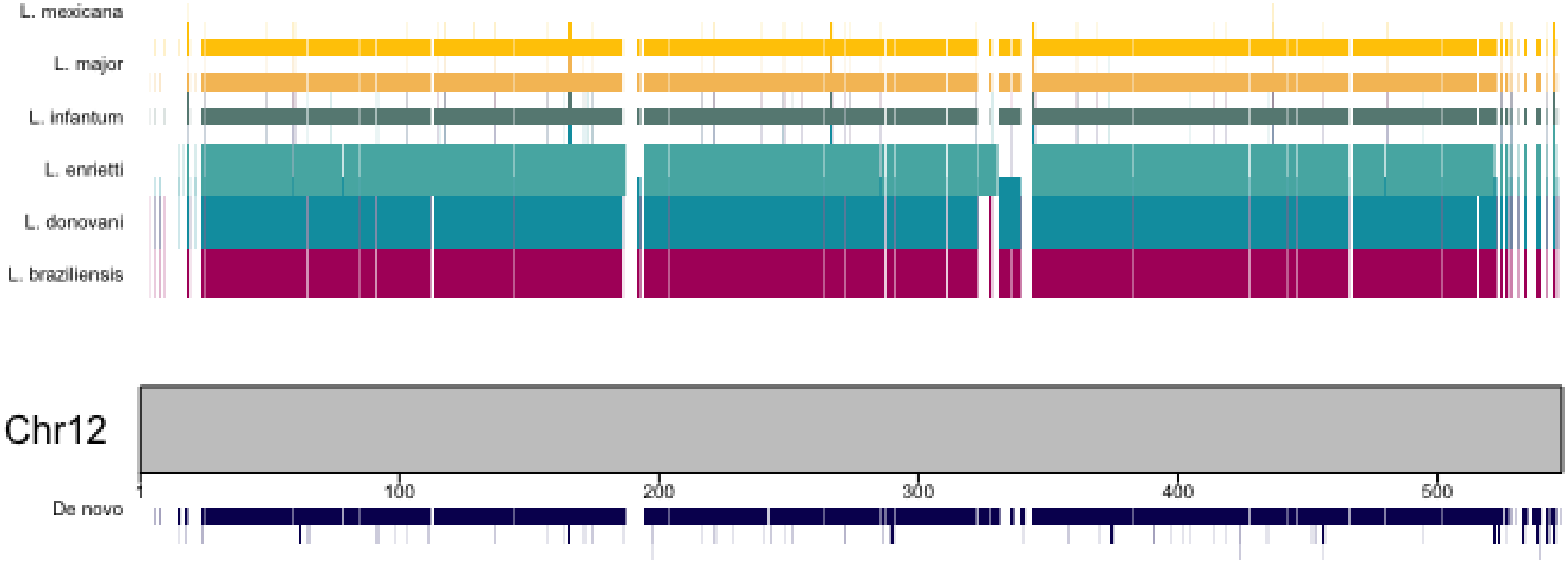
L. mexicana
L. major
L. infantum
L. enrietti
L. donovani
L. braziliensis
Chr12
1
100
200
300
400
500
De novo

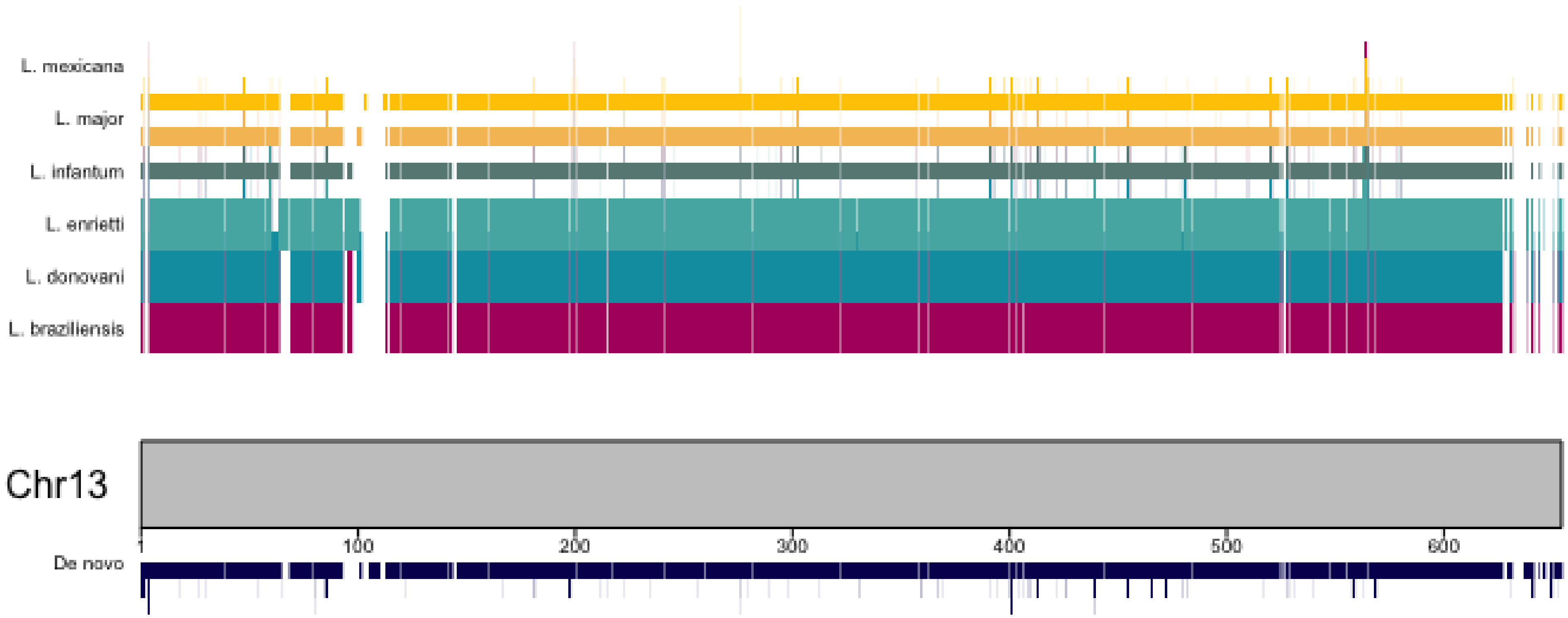

L. mexicana
L. major
L. infantum
L. enrietti
L. donovani
L. braziliensis
Chr13
1
100
200
300
400
500
600
De novo

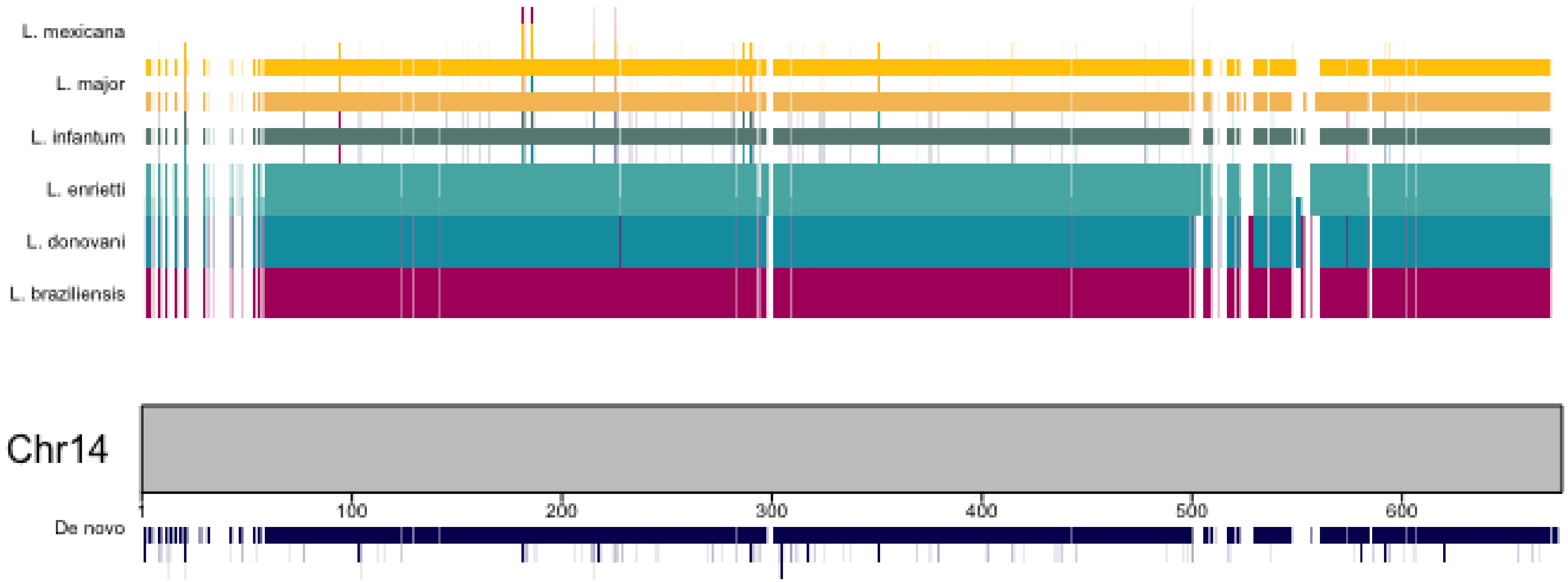

L. mexicana
L. major
L. infantum
L. enrietti
L. donovani
L. braziliensis
Chr14
1
100
200
300
400
500
600
De novo

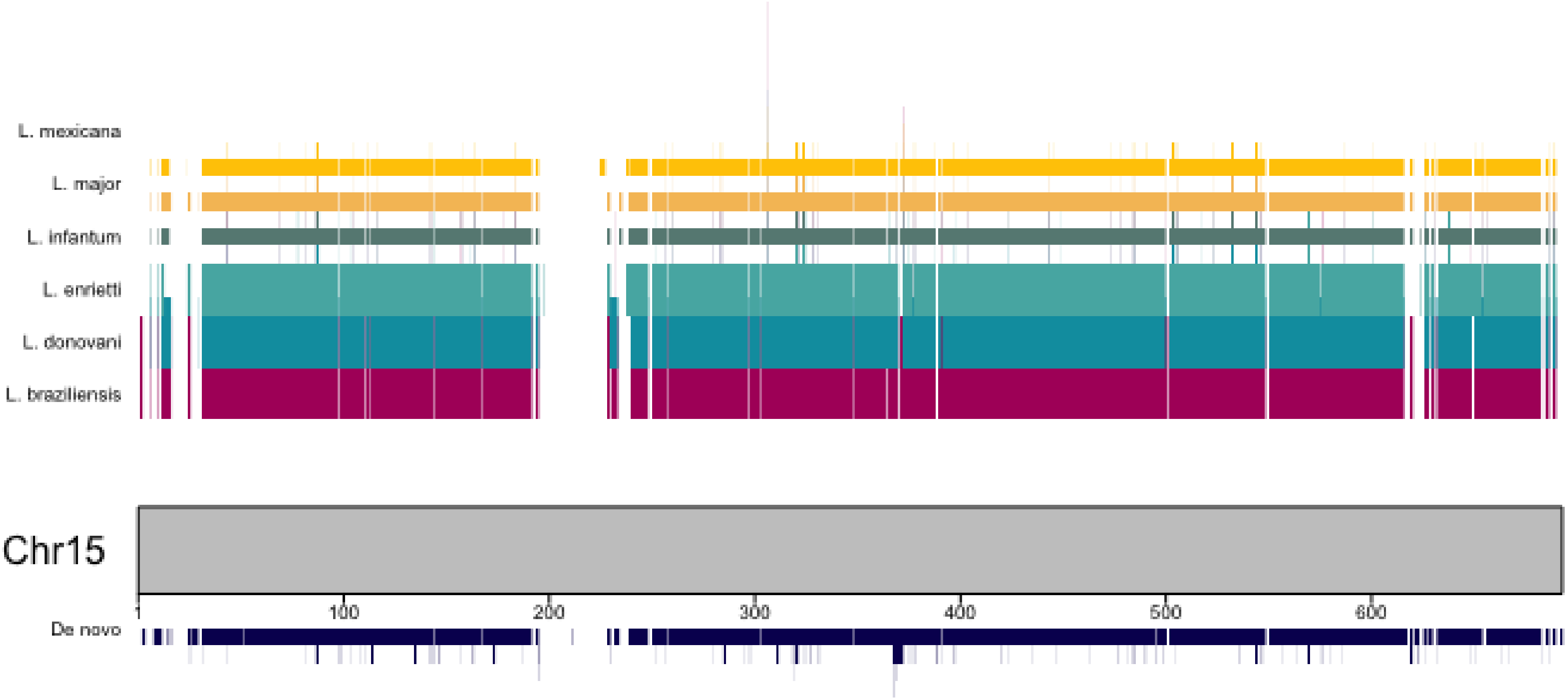

L. mexicana
L. major
L. infantum
L. enriettii
L. donovani
L. braziliensis
Chr15
1
100
200
300
400
500
600
De novo

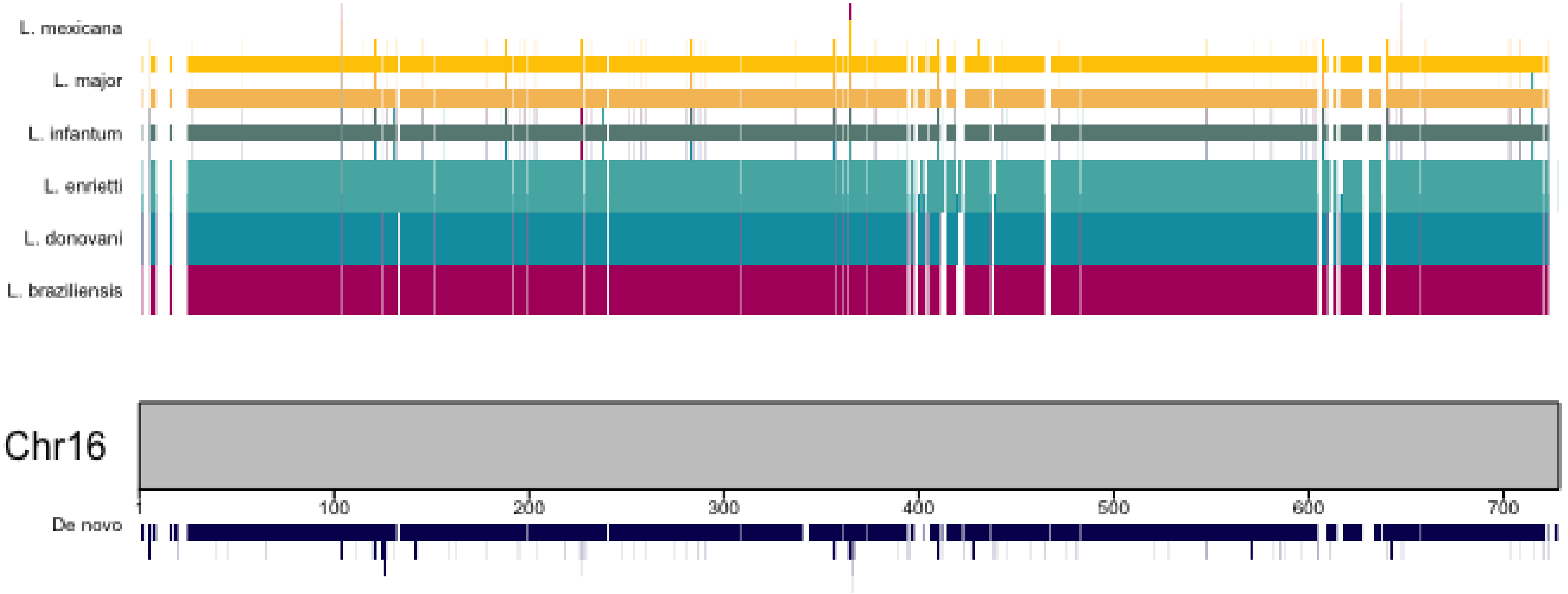

L. mexicana
L. major
L. infantum
L. enrietti
L. donovani
L. braziliensis
Chr16
1
100
200
300
400
500
600
700
De novo

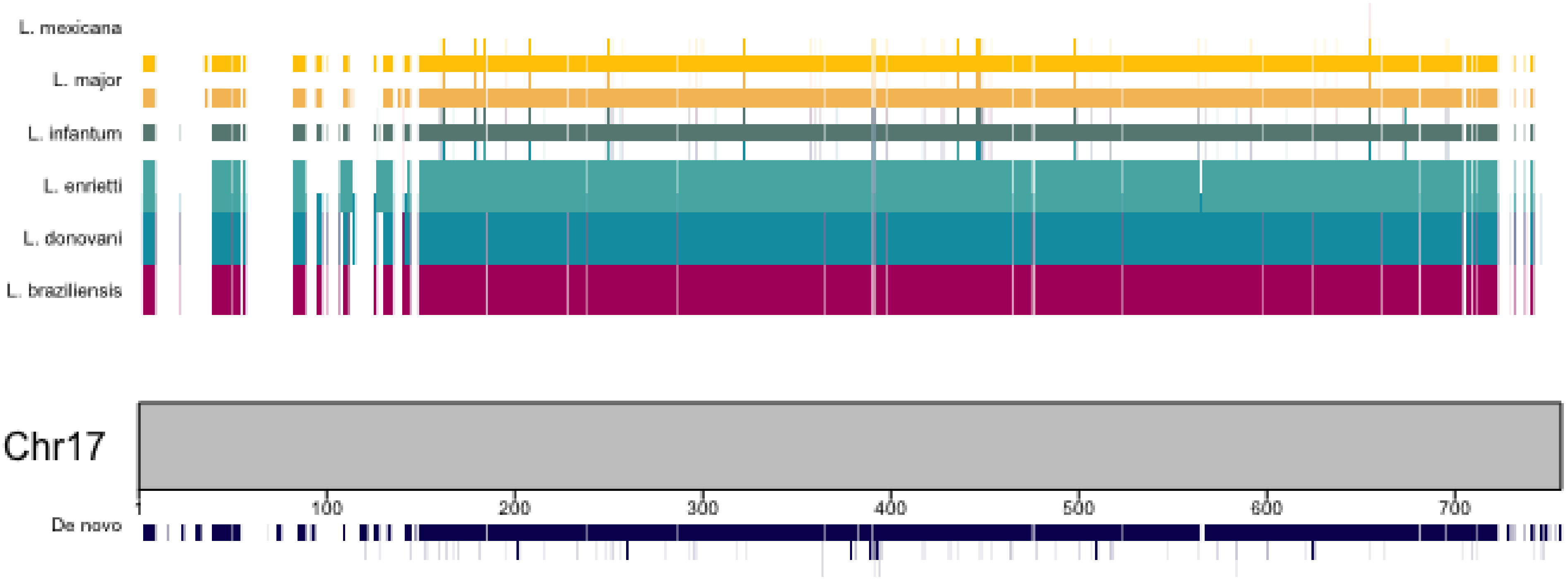

L. mexicana
L. major
L. infantum
L. enrietti
L. donovani
L. braziliensis
Chr17
1
100
200
300
400
500
600
700
De novo

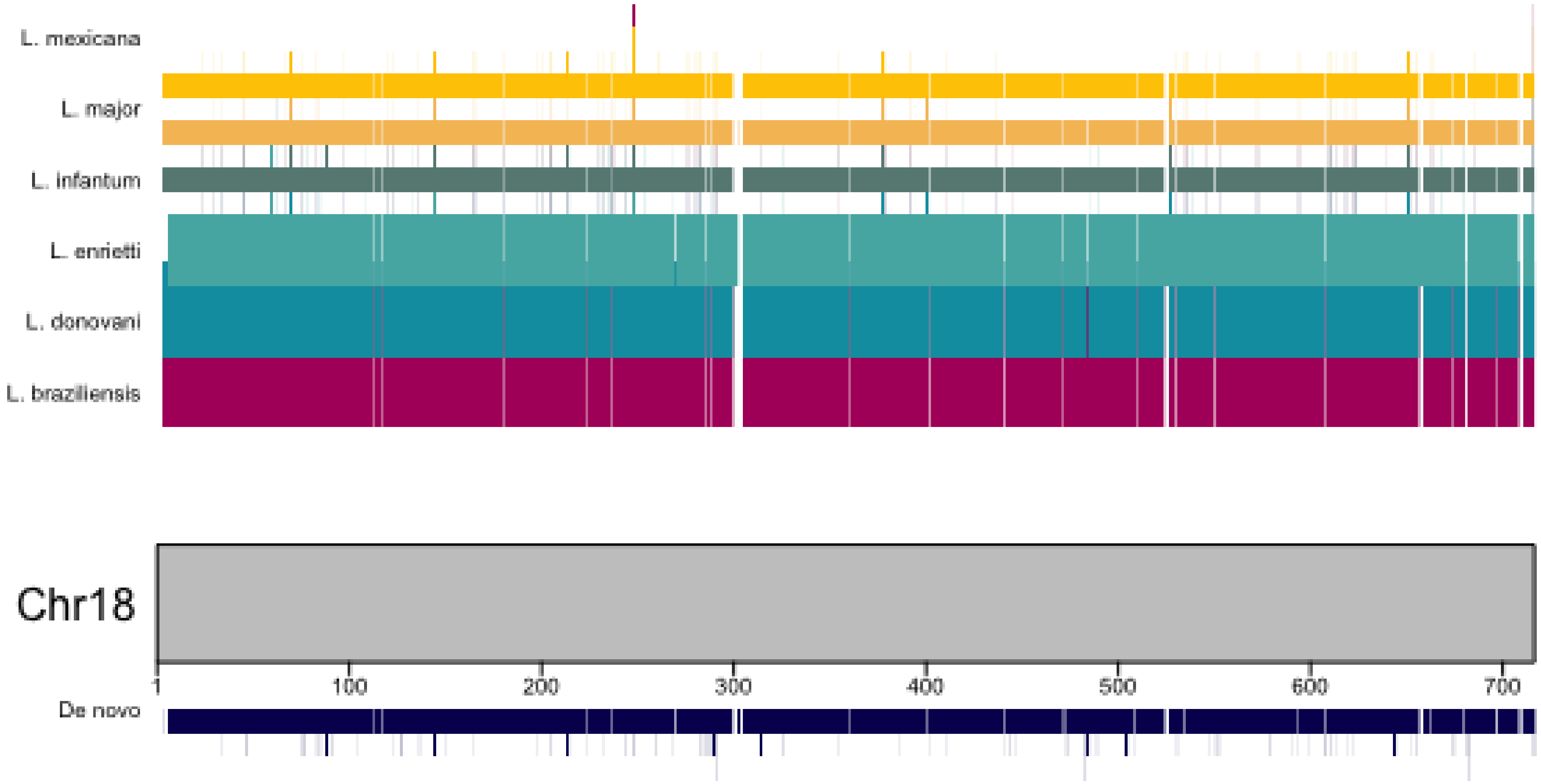
L. mexicana
L. major
L. infantum
L. enriettii
L. donovani
L. braziliensis
Chr18
1
100
200
300
400
500
600
700
De novo

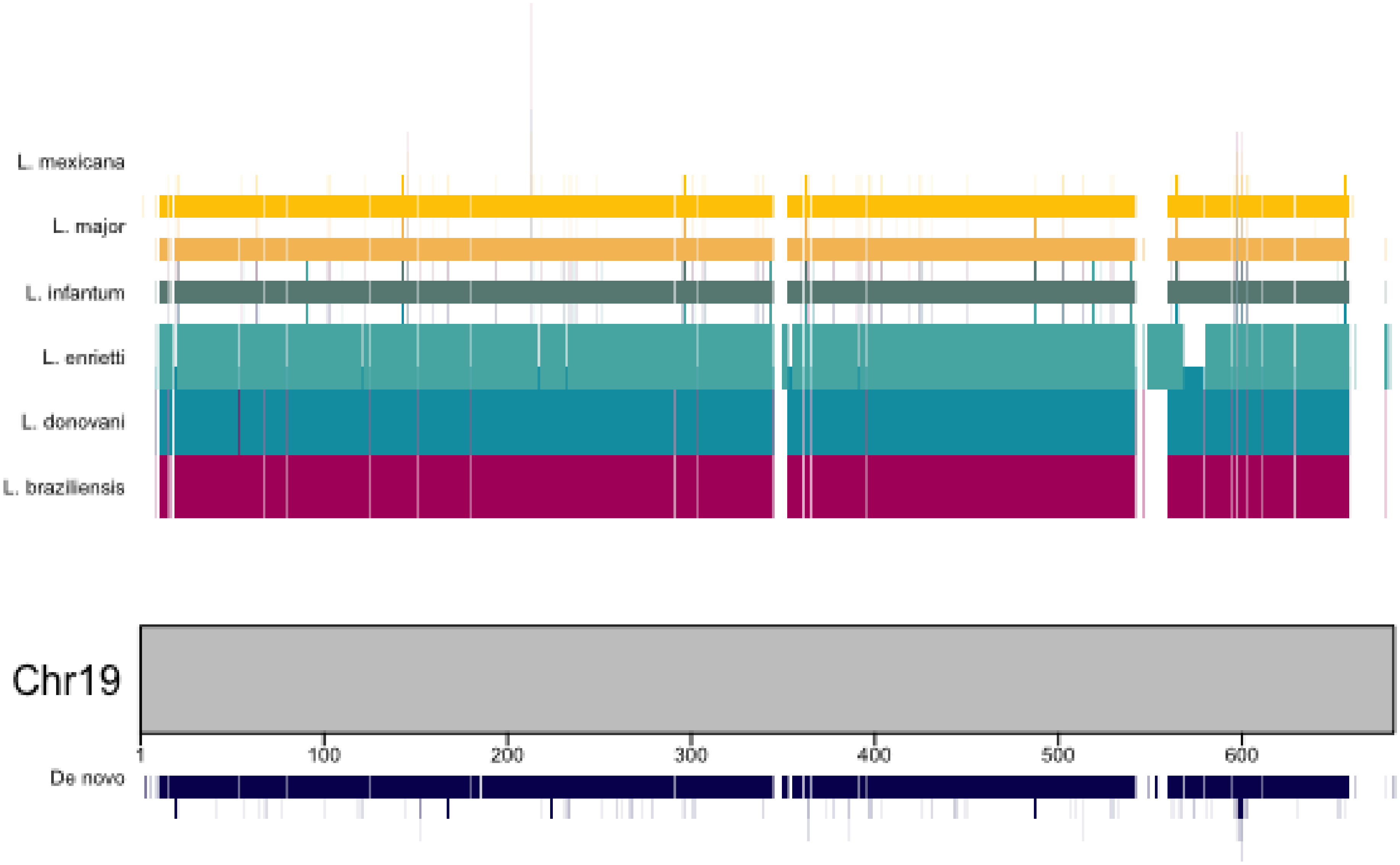
L. mexicana
L. major
L. infantum
L. enrietti
L. donovani
L. braziliensis
Chr19
1
100
200
300
400
500
600
De novo

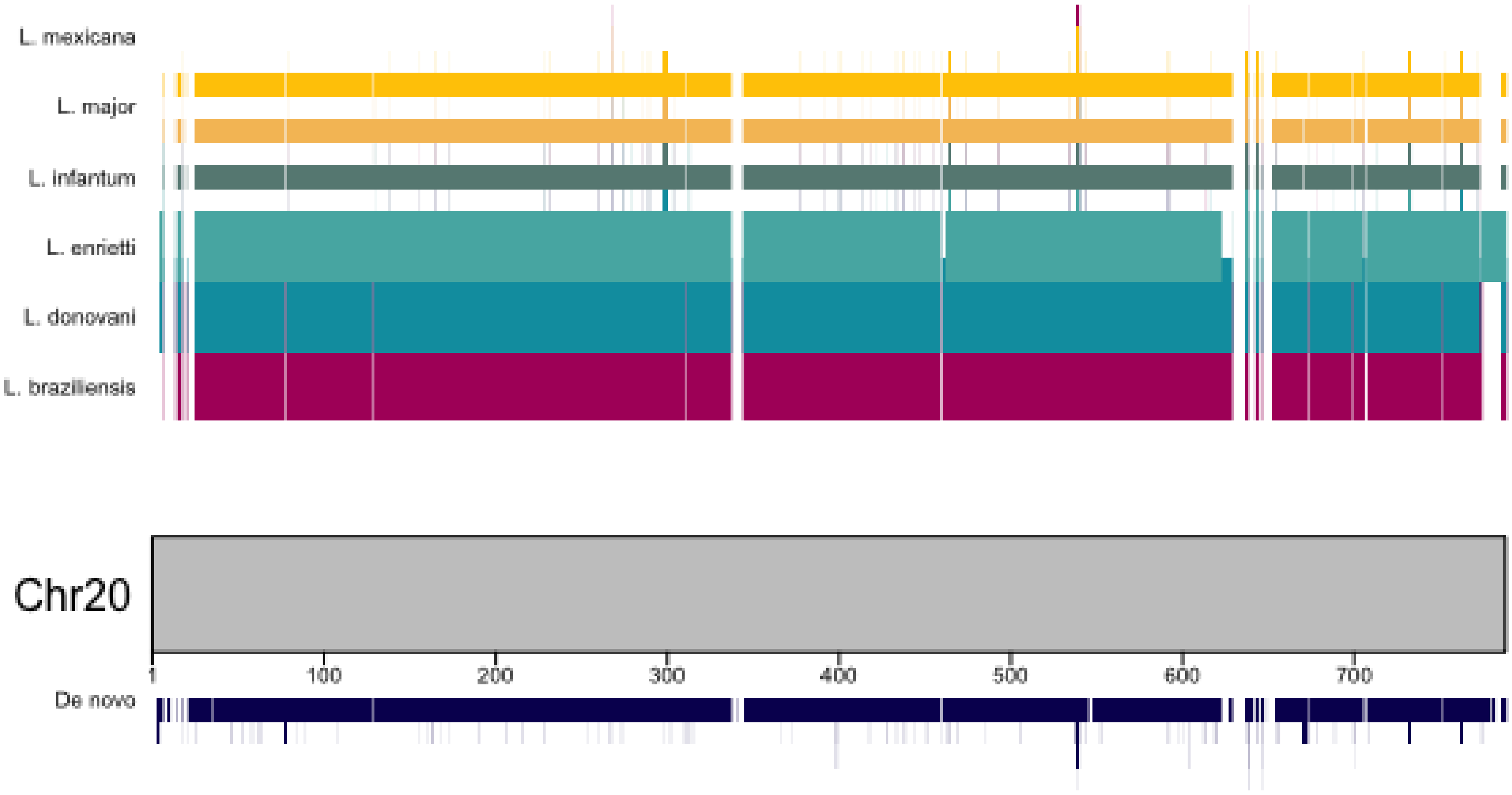

L. mexicana
L. major
L. infantum
L. enriettii
L. donovani
L. braziliensis
Chr20
1
100
200
300
400
500
600
700
De novo

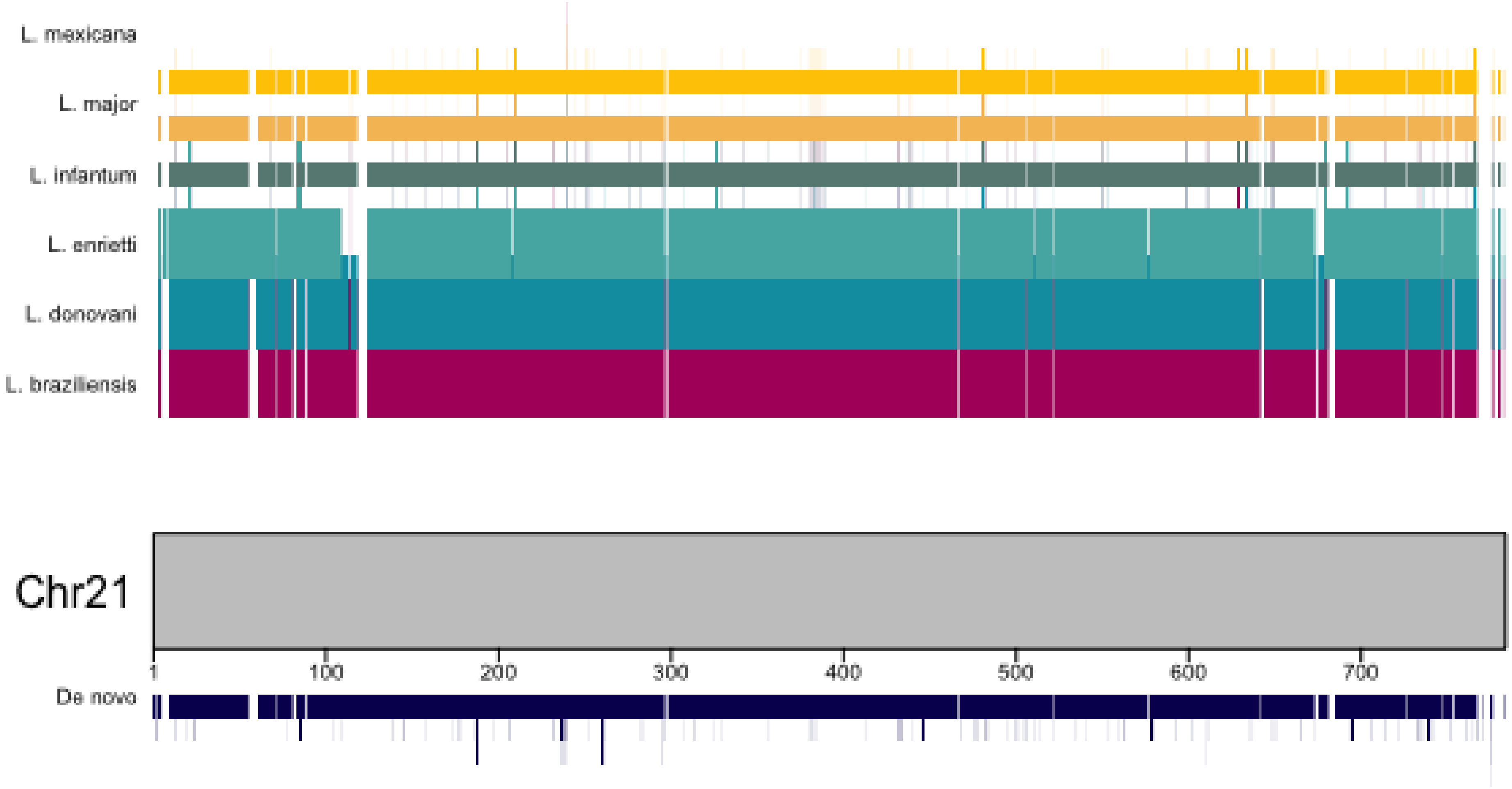
L. mexicana
L. major
L. infantum
L. enrietti
L. donovani
L. braziliensis
Chr21
1
100
200
300
400
500
600
700
De novo

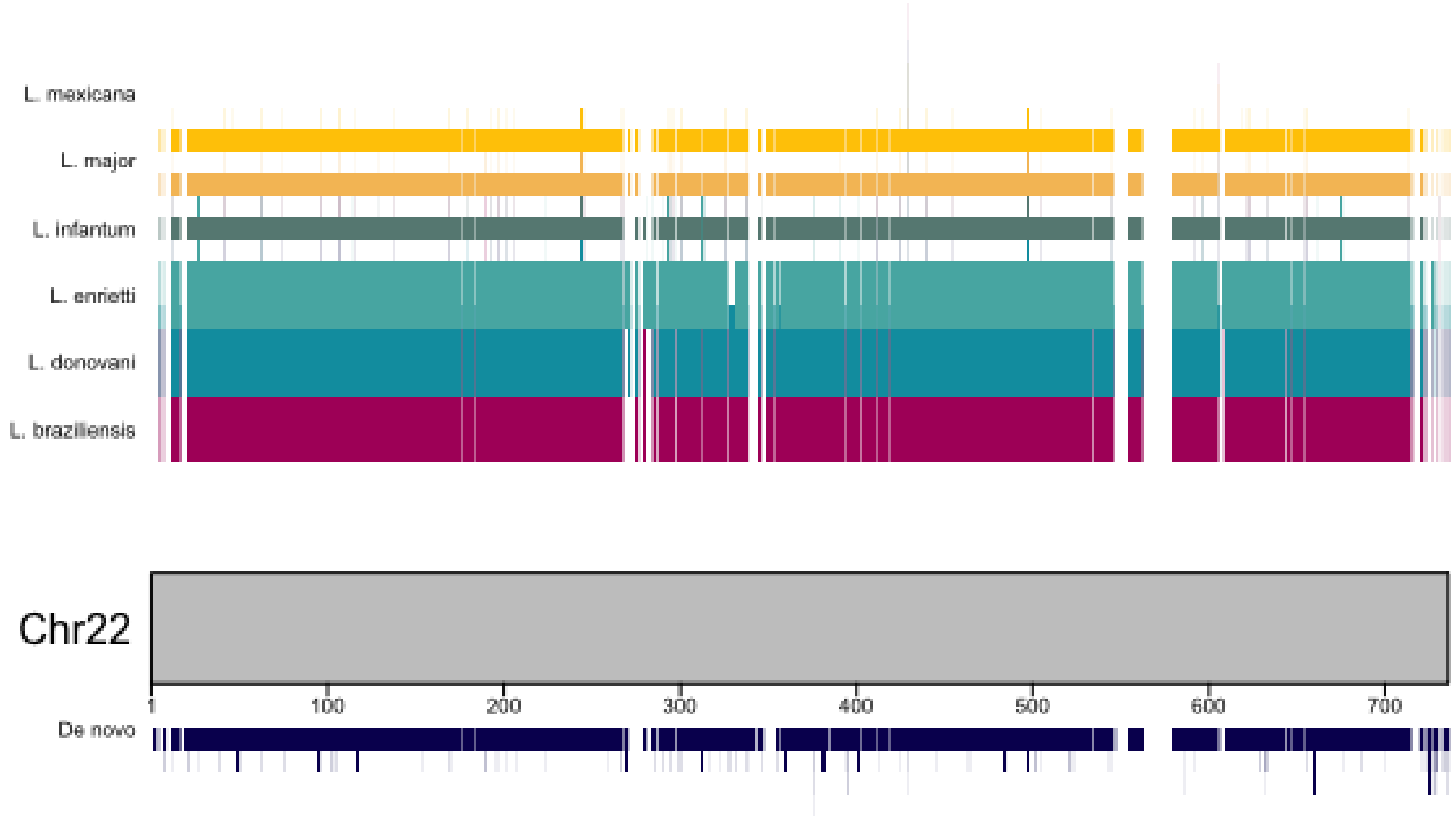
L. mexicana
L. major
L. infantum
L. enrietti
L. donovani
L. braziliensis
Chr22
1
100
200
300
400
500
600
700
De novo

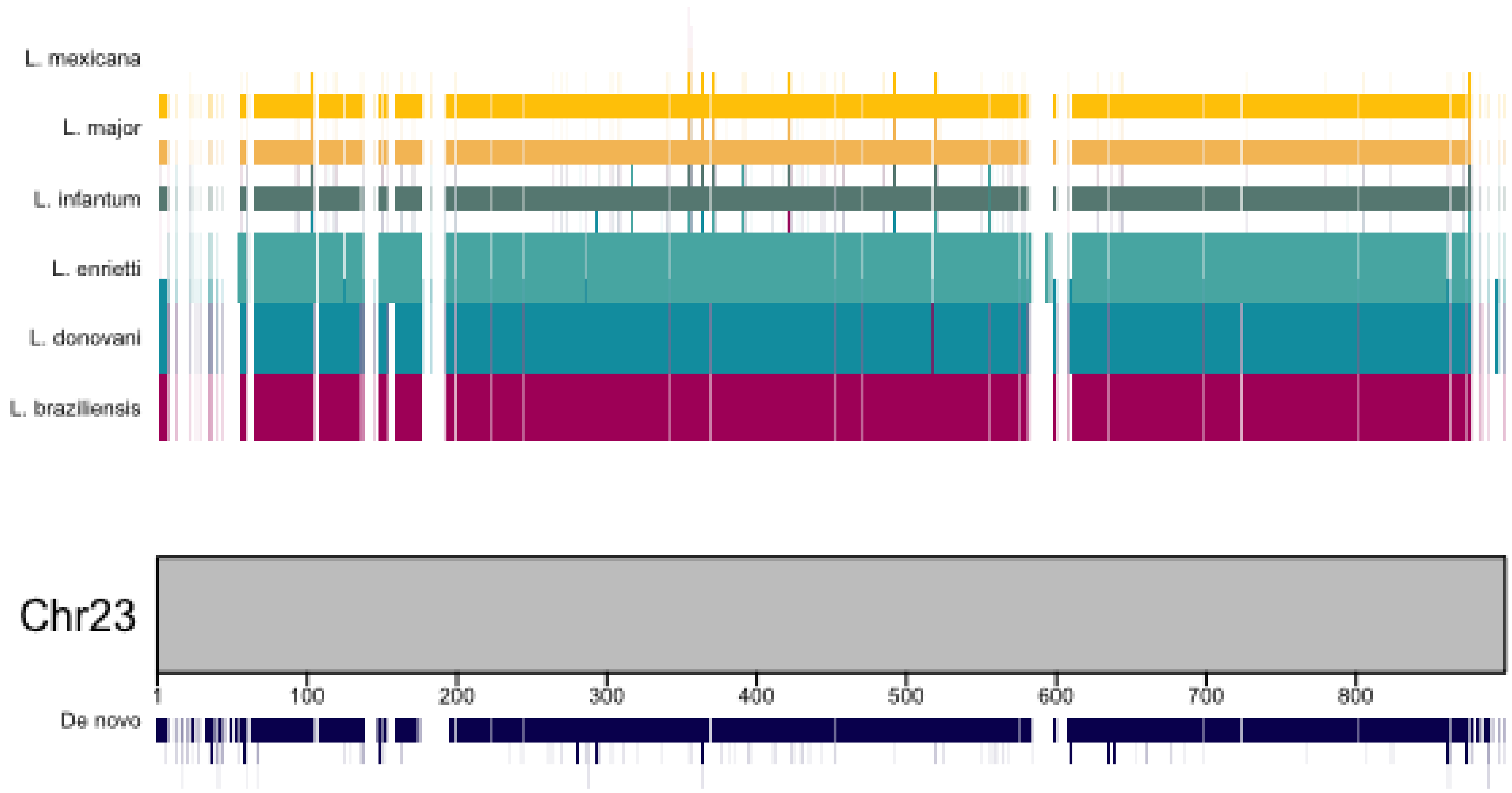

L. mexicana
L. major
L. infantum
L. enrietti
L. donovani
L. braziliensis
Chr23
1
100
200
300
400
500
600
700
800
De novo

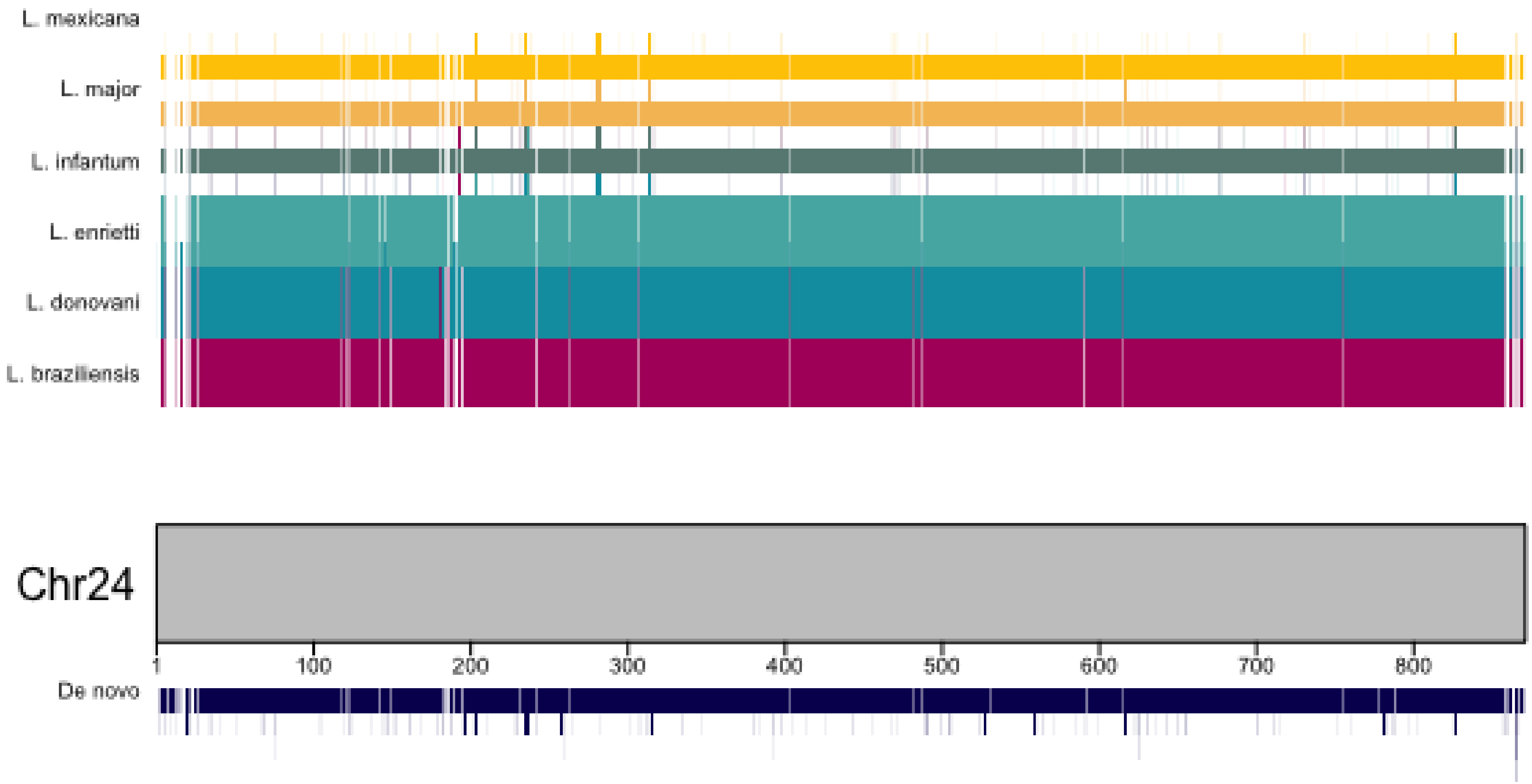

L. mexicana
L. major
L. infantum
L. enrietti
L. donovani
L. braziliensis
Chr24
1
100
200
300
400
500
600
700
800
De novo

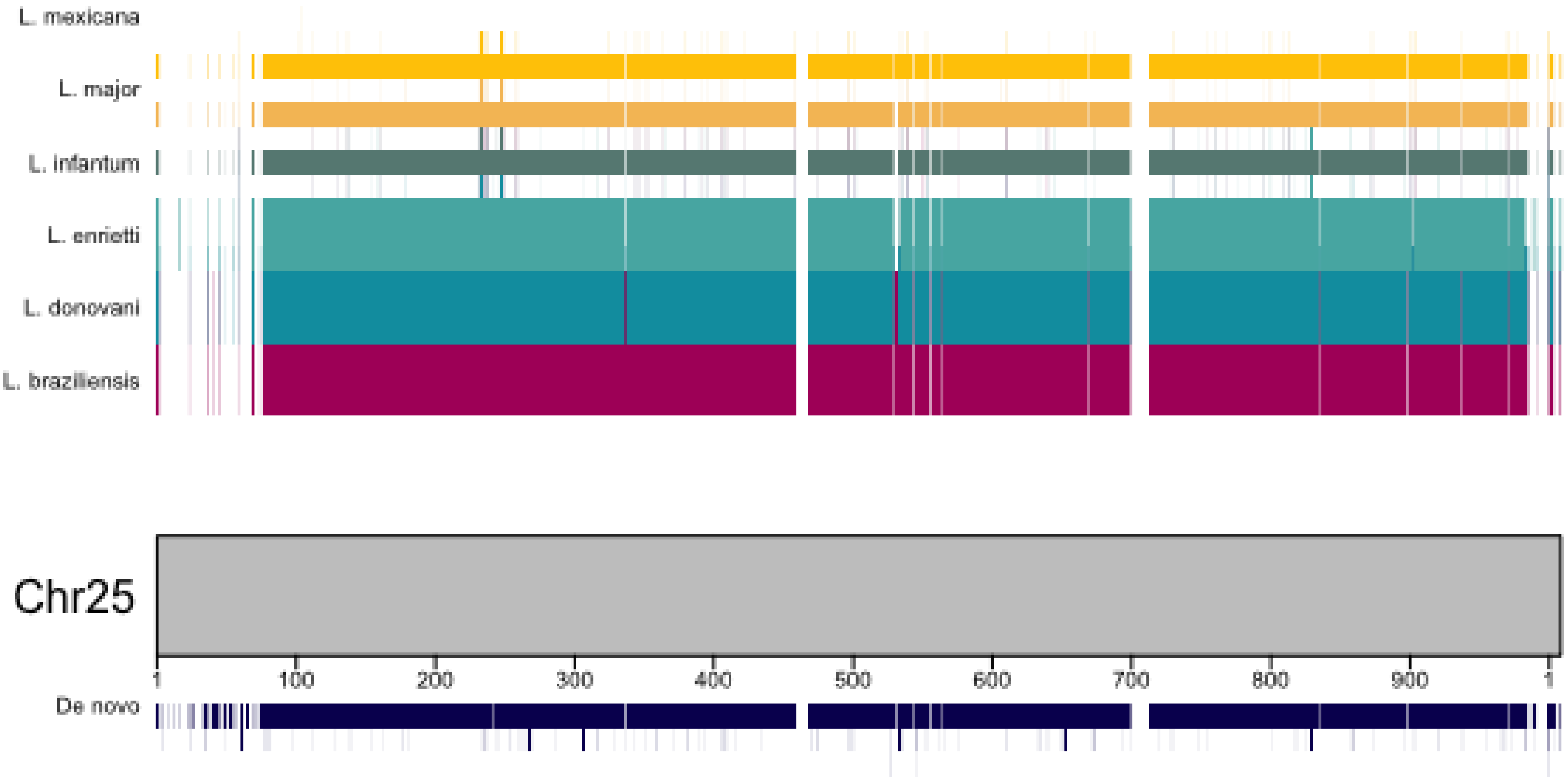

L. mexicana
L. major
L. infantum
L. enrietti
L. donovani
L. braziliensis
Chr25
1
100
200
300
400
500
600
700
800
900
1
De novo

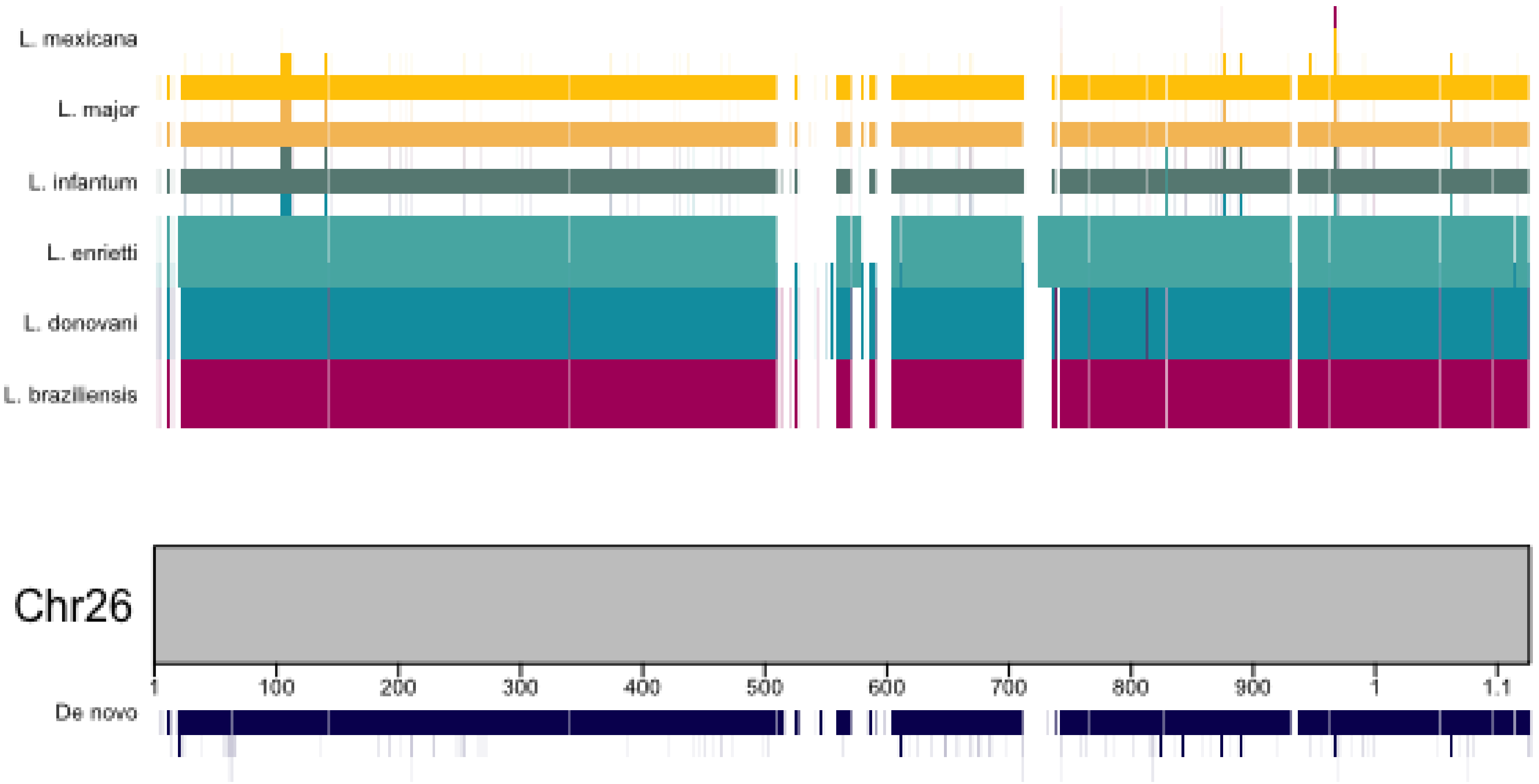

L. mexicana
L. major
L. infantum
L. enrietti
L. donovani
L. braziliensis
Chr26
1
100
200
300
400
500
600
700
800
900
1
1.1
De novo

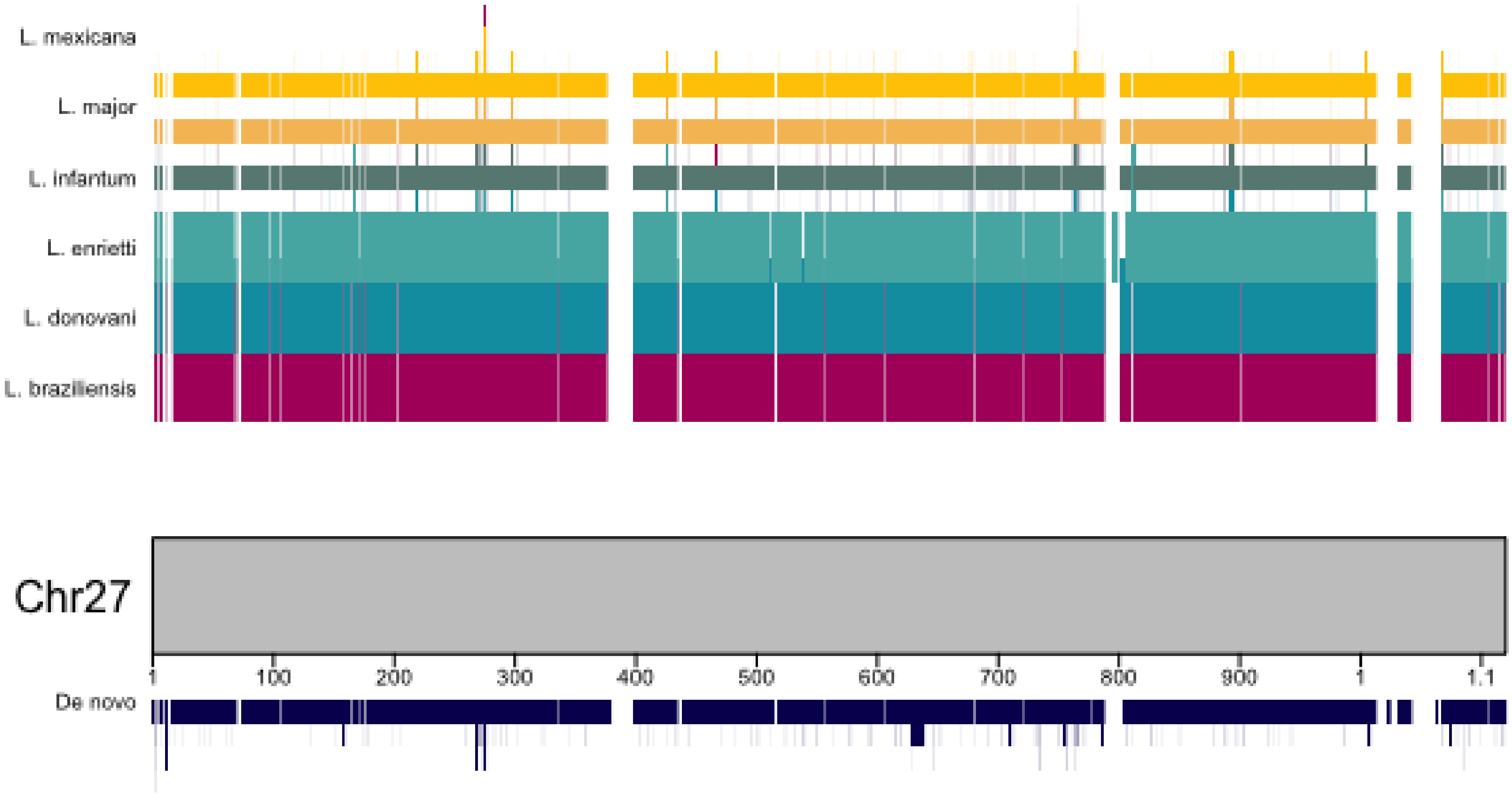

L. mexicana
L. major
L. infantum
L. enrietti
L. donovani
L. braziliensis
Chr27
1
100
200
300
400
500
600
700
800
900
1
1.1
De novo

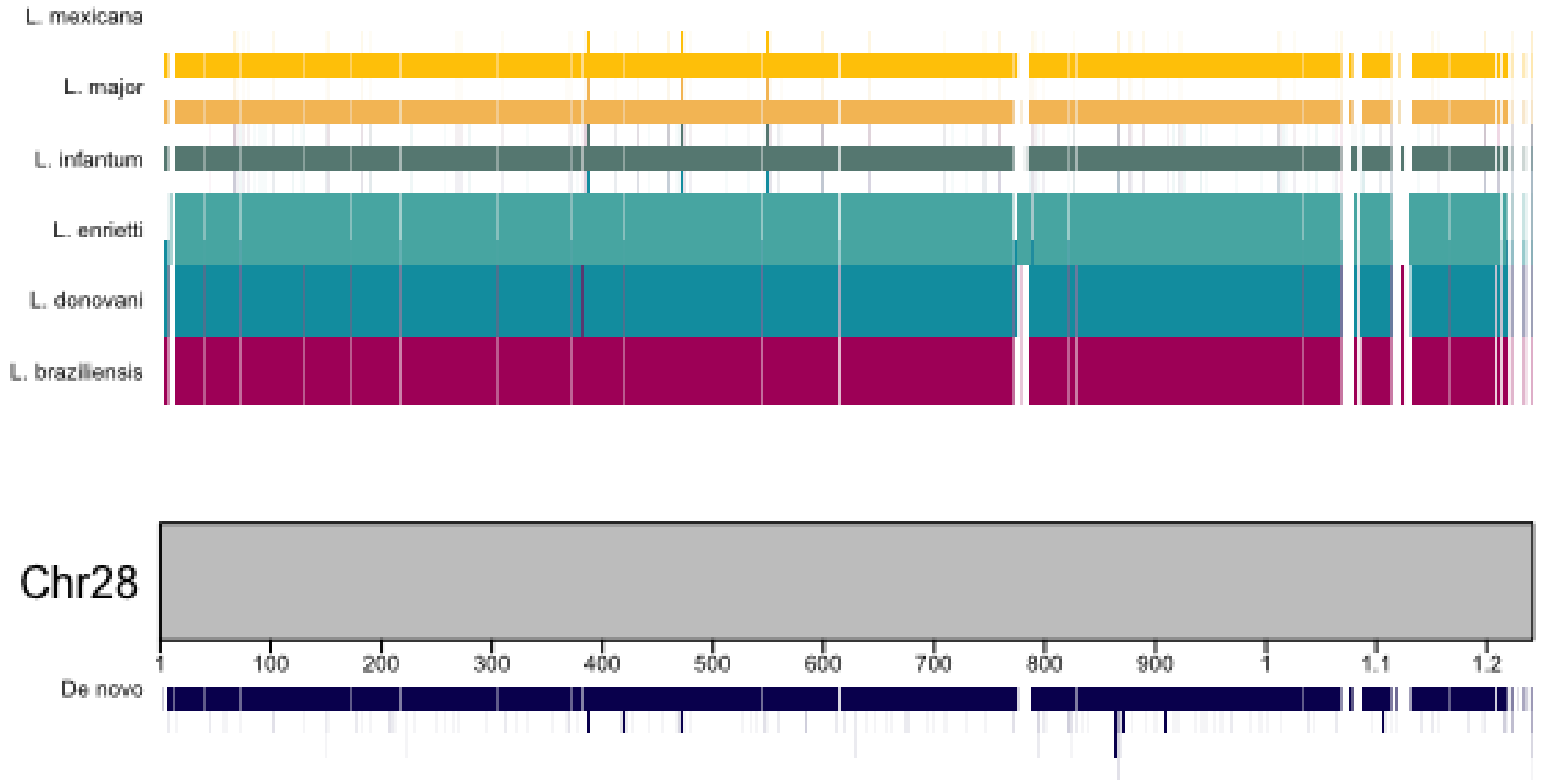
L. mexicana
L. major
L. infantum
L. enrietti
L. donovani
L. braziliensis
Chr28
1
100
200
300
400
500
600
700
800
900
1
1.1
1.2
De novo

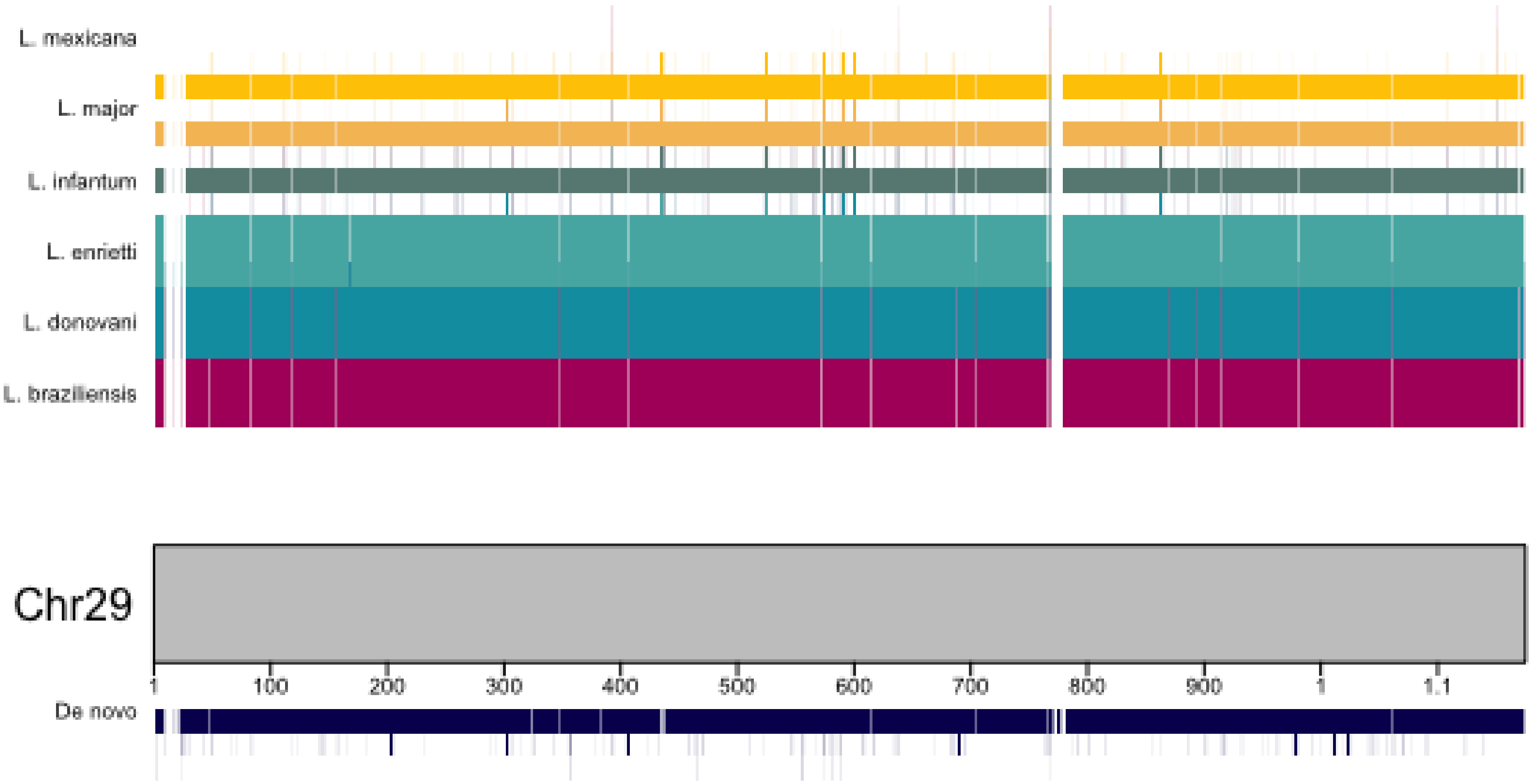

L. mexicana
L. major
L. infantum
L. enriettii
L. donovani
L. braziliensis
Chr29
1
100
200
300
400
500
600
700
800
900
1
1.1
De novo

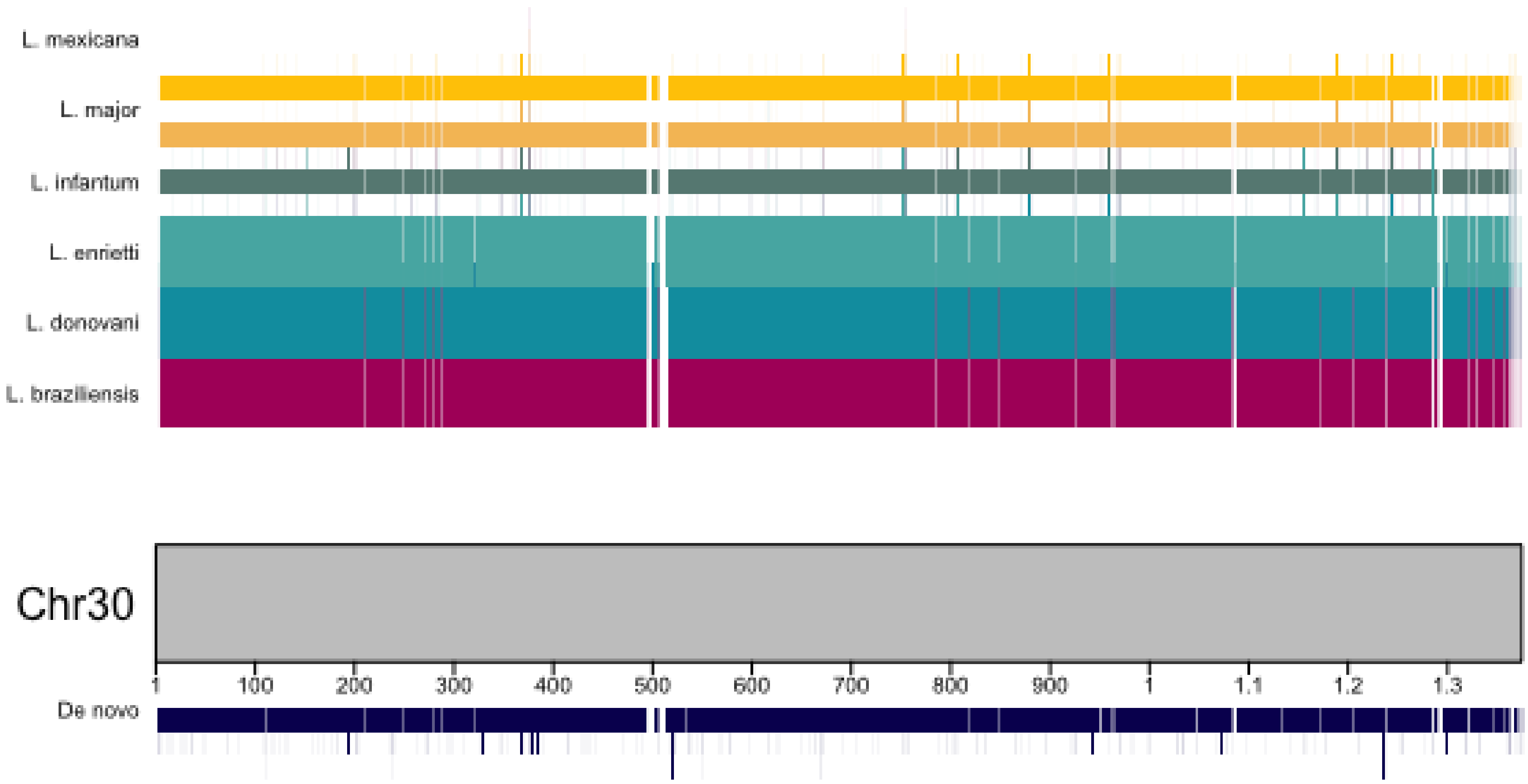

L. mexicana
L. major
L. infantum
L. enriettii
L. donovani
L. braziliensis
Chr30
1
100
200
300
400
500
600
700
800
900
1
1.1
1.2
1.3
De novo

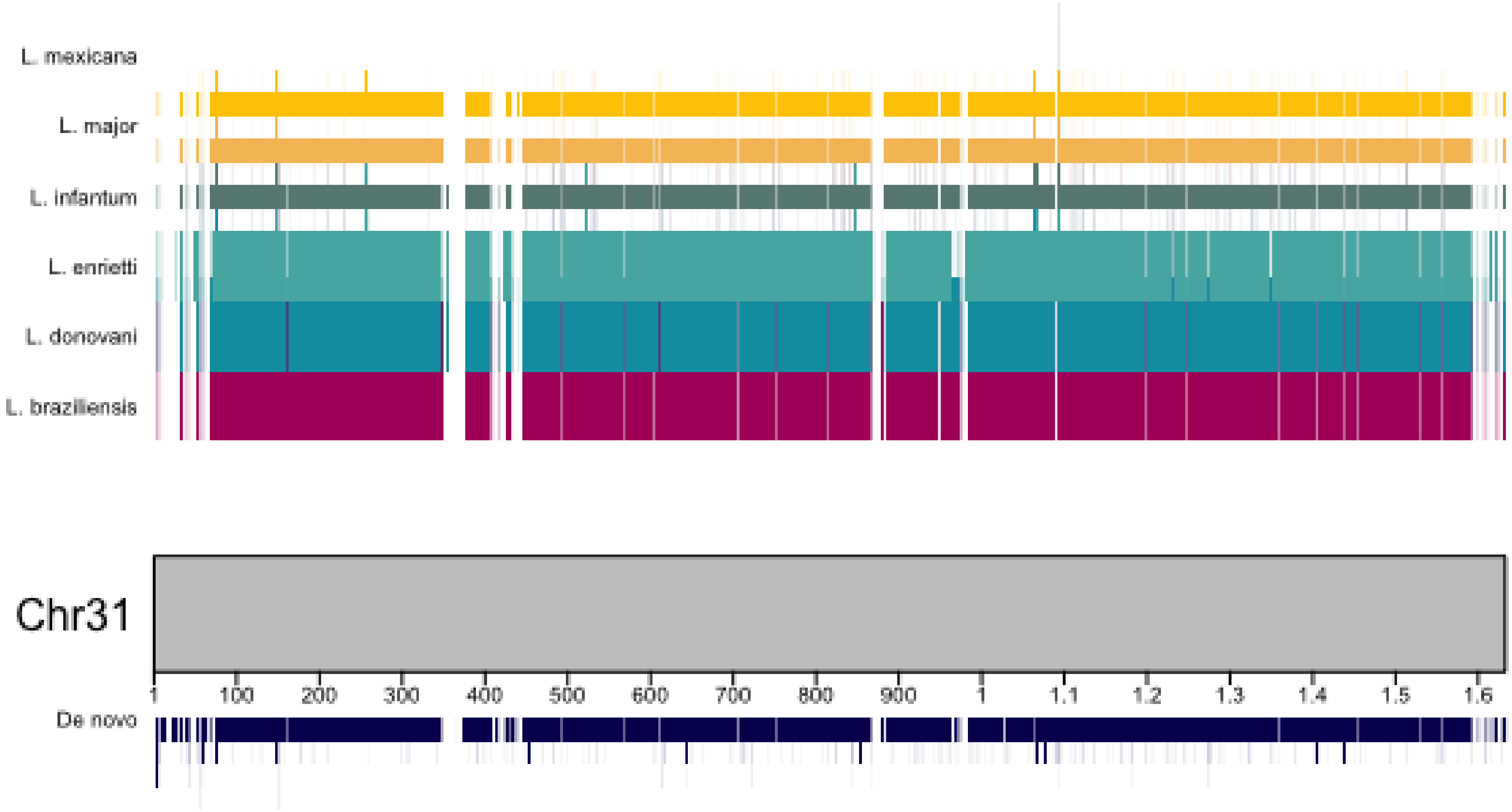
L. mexicana
L. major
L. infantum
L. enrietti
L. donovani
L. braziliensis
Chr31
1
100
200
300
400
500
600
700
800
900
1
1.1
1.2
1.3
1.4
1.5
1.6
De novo

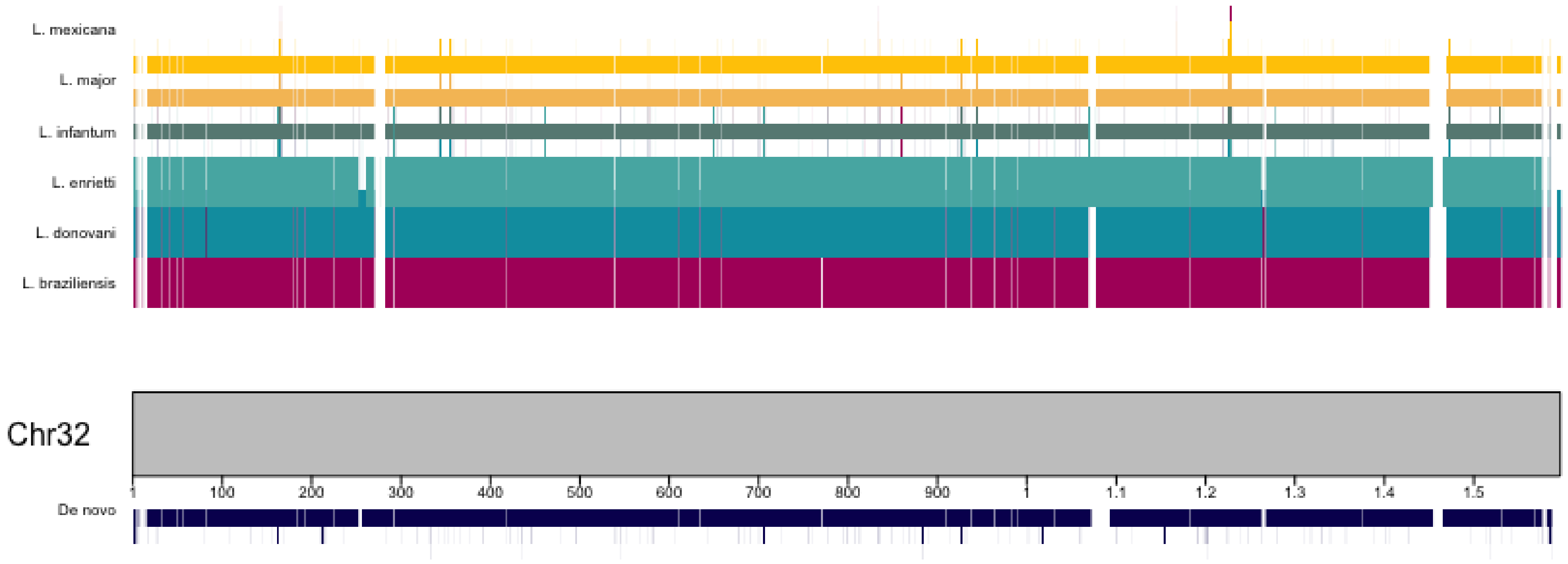

L. mexicana
L. major
L. infantum
L. enrietti
L. donovani
L. braziliensis
Chr32
1
100
200
300
400
500
600
700
800
900
1
1.1
1.2
1.3
1.4
1.5
De novo

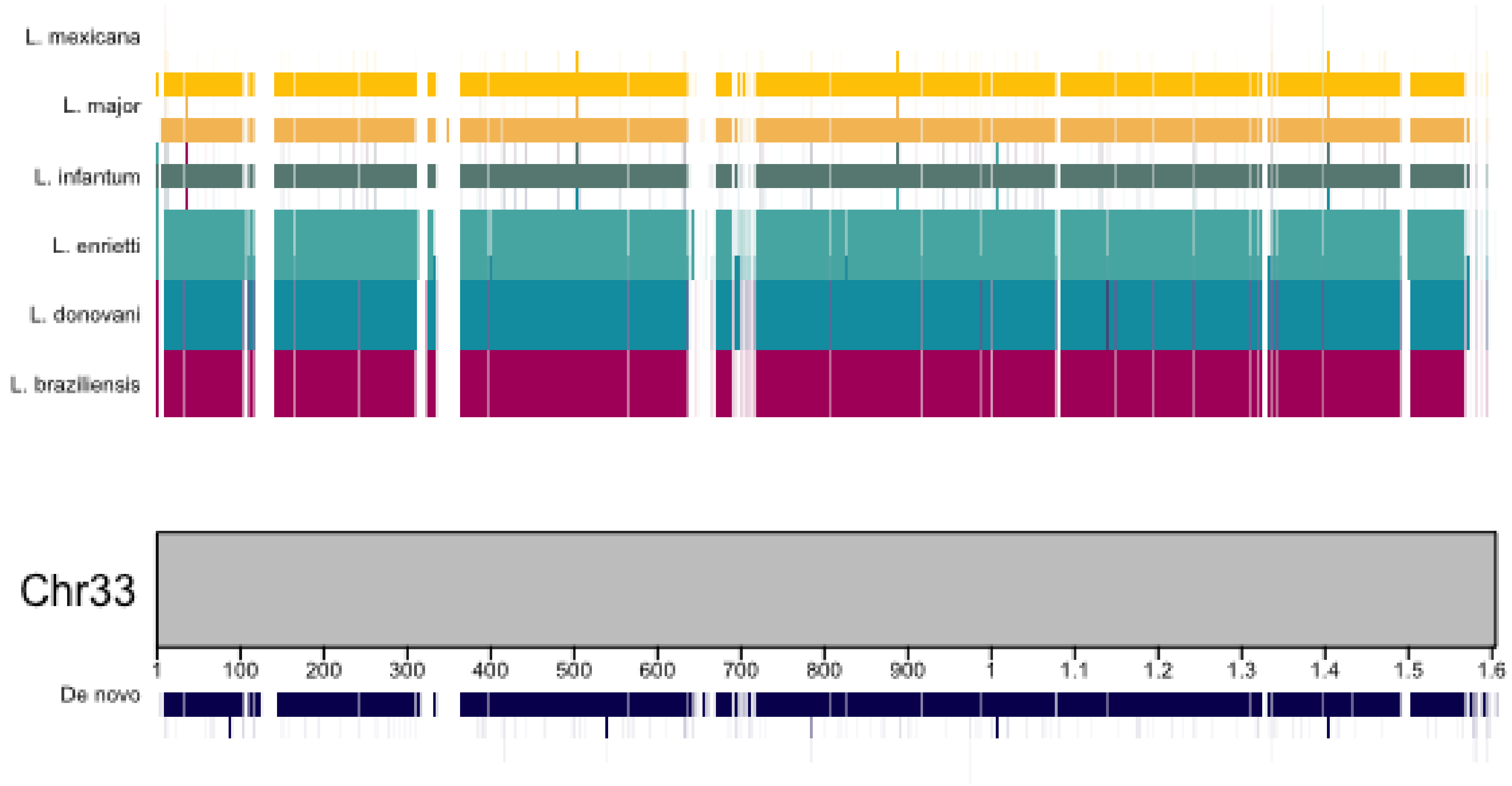

L. mexicana
L. major
L. infantum
L. enriettii
L. donovani
L. braziliensis
Chr33
1
100
200
300
400
500
600
700
800
900
1
1.1
1.2
1.3
1.4
1.5
1.6
De novo

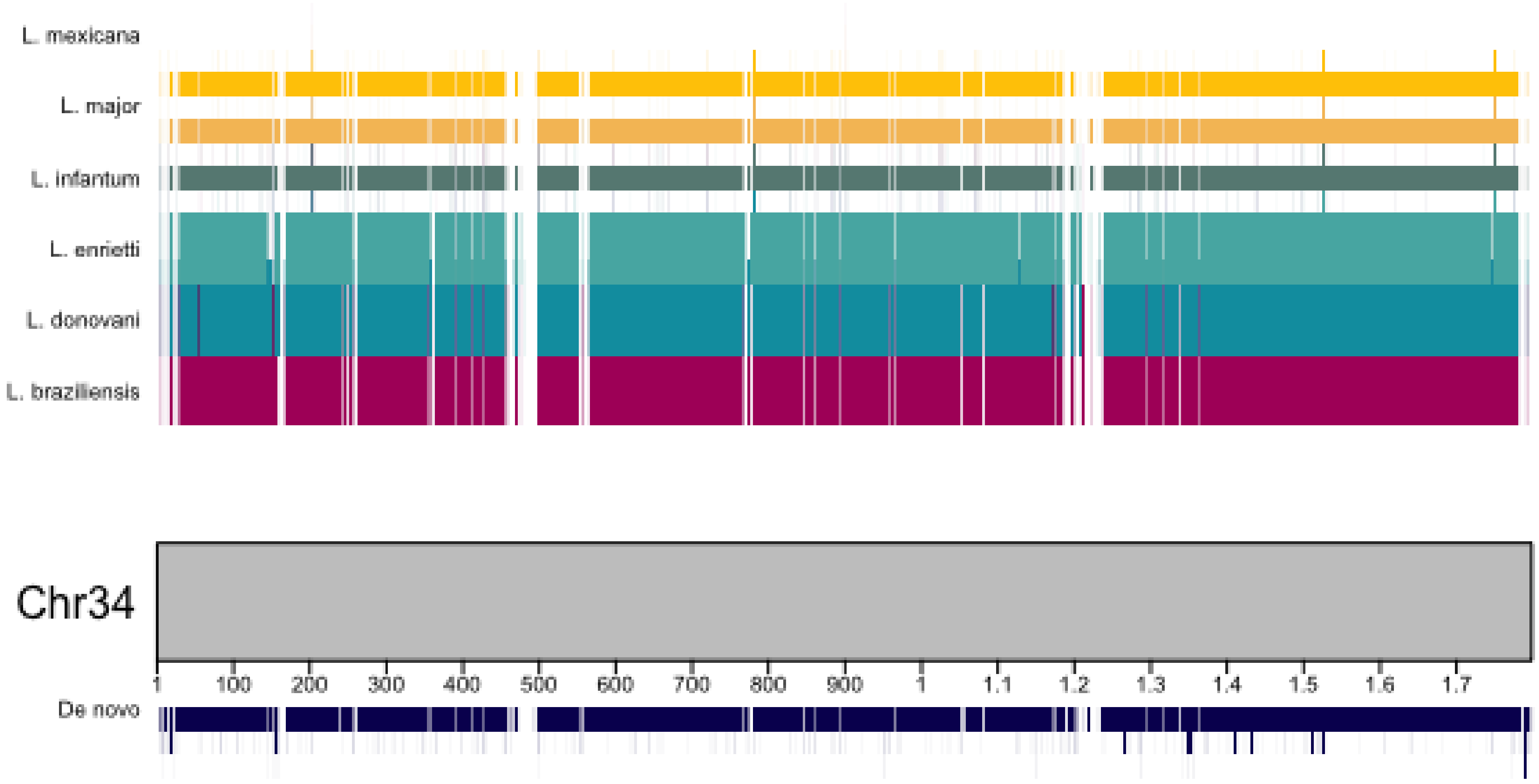

L. mexicana
L. major
L. infantum
L. enrietti
L. donovani
L. braziliensis
Chr34
1
100
200
300
400
500
600
700
800
900
1
1.1
1.2
1.3
1.4
1.5
1.6
1.7
De novo

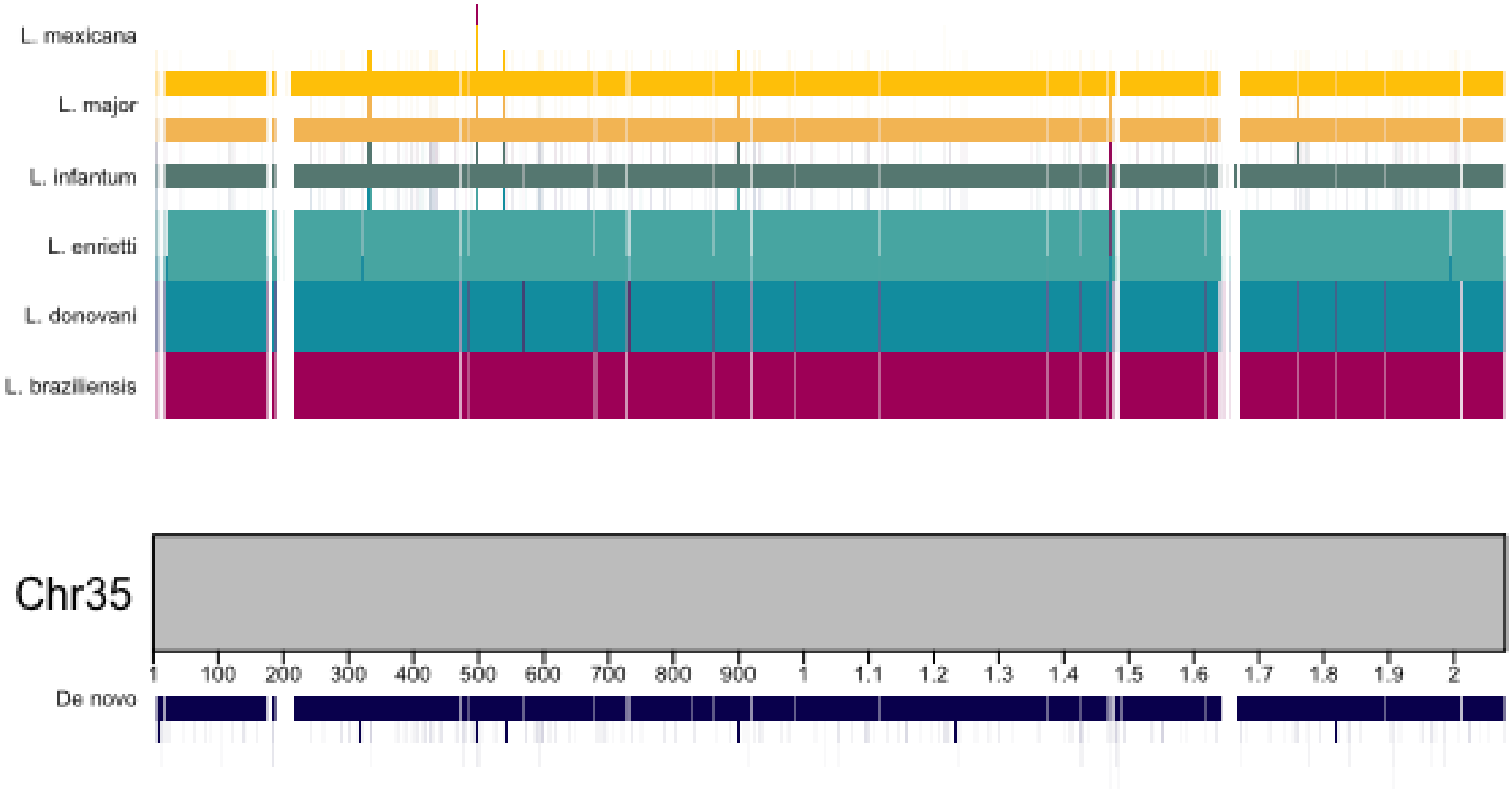

L. mexicana
L. major
L. infantum
L. enrietti
L. donovani
L. braziliensis
Chr35
1
100
200
300
400
500
600
700
800
900
1
1.1
1.2
1.3
1.4
1.5
1.6
1.7
1.8
1.9
2
De novo

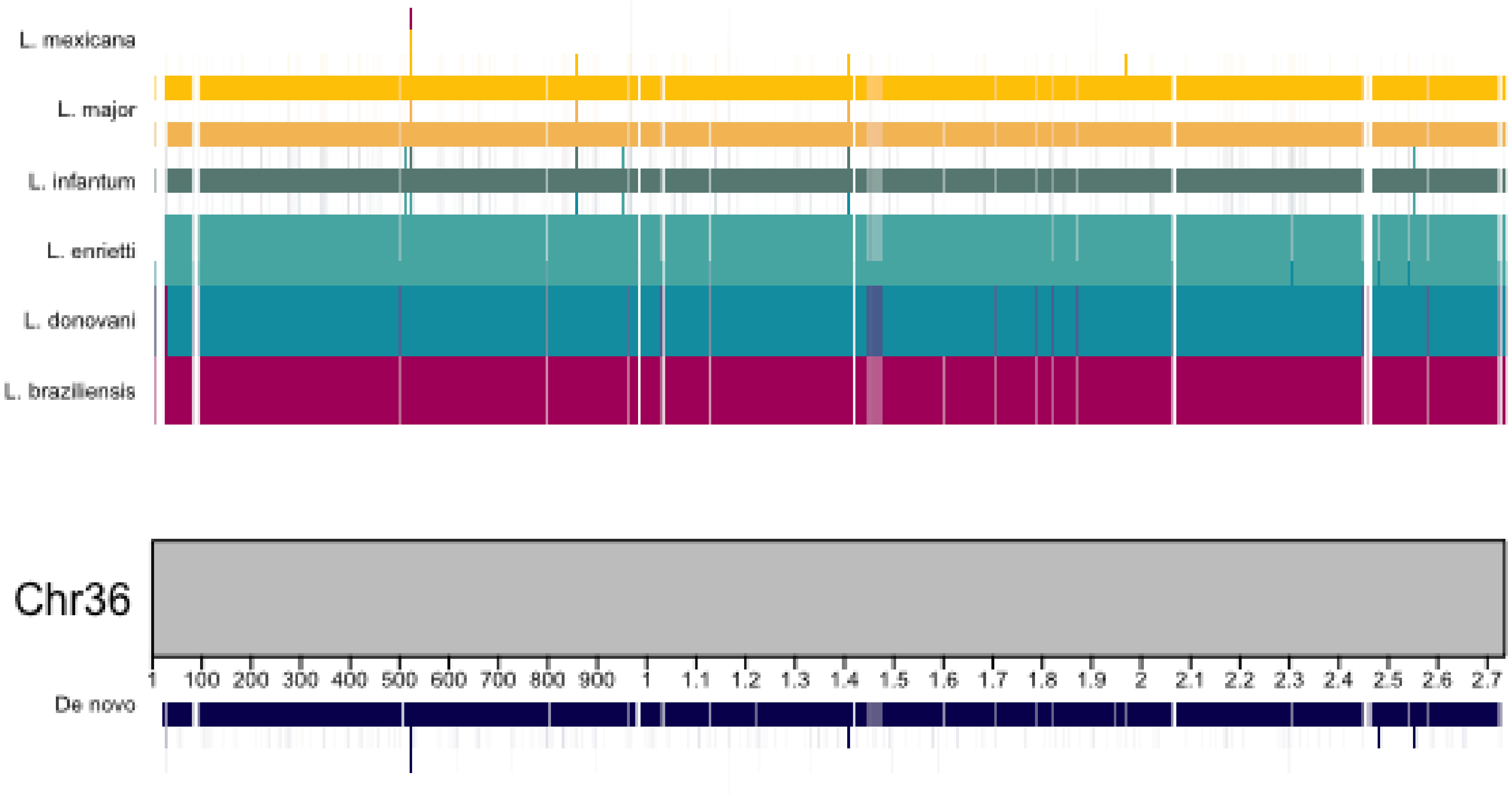

L. mexicana
L. major
L. infantum
L. enriettii
L. donovani
L. braziliensis
Chr36
1
100
200
300
400
500
600
700
800
900
1
1.1
1.2
1.3
1.4
1.5
1.6
1.7
1.8
1.9
2
2.1
2.2
2.3
2.4
2.5
2.6
2.7
De novo
