# Supplementary figures and images for: Making the Most of Its Short Reads: A Bioinformatics Workflow for Analysing the Short-Read-Only Data of Leishmania orientalis (Formerly Named Leishmania siamensis) Isolate PCM2 in Thailand
Source: Biology (Basel). 2022 Aug 26;11(9):1272. doi: 10.3390/biology11091272 (PMC9495971; doi:10.3390/biology11091272)

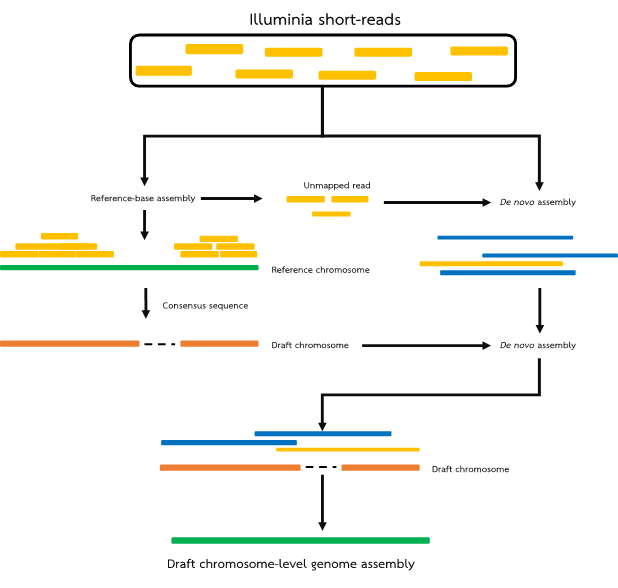

Supplement: Supplementary file 1 [file biology-11-01272-s001.zip › Figure S2.png]
